# The genetic profile of Leber congenital amaurosis in an Australian cohort

**DOI:** 10.1002/mgg3.321

**Published:** 2017-08-22

**Authors:** Jennifer A. Thompson, John N. De Roach, Terri L. McLaren, Hannah E. Montgomery, Ling H. Hoffmann, Isabella R. Campbell, Fred K. Chen, David A. Mackey, Tina M. Lamey

**Affiliations:** ^1^ Australian Inherited Retinal Disease Registry and DNA Bank Department of Medical Technology and Physics Sir Charles Gairdner Hospital Perth Western Australia Australia; ^2^ Centre for Ophthalmology and Visual Science The University of Western Australia Crawley Western Australia Australia; ^3^ Lions Eye Institute Nedlands Western Australia Australia; ^4^ Department of Ophthalmology Royal Perth Hospital Perth Western Australia Australia

**Keywords:** Genetic variants, inherited retinal dystrophies, Leber congenital amaurosis, next‐generation sequencing, personalized therapies, retinal disease

## Abstract

**Background:**

Leber congenital amaurosis (LCA) is a severe visual impairment responsible for infantile blindness, representing ~5% of all inherited retinal dystrophies. LCA encompasses a group of heterogeneous disorders, with 24 genes currently implicated in pathogenesis. Such clinical and genetic heterogeneity poses great challenges for treatment, with personalized therapies anticipated to be the best treatment candidates. Unraveling the individual genetic etiology of disease is a prerequisite for personalized therapies, and could identify potential treatment candidates, inform patient management, and discriminate syndromic forms of disease.

**Methods:**

We have genetically analyzed 45 affected and 82 unaffected individuals from 34 unrelated LCA pedigrees using predominantly next‐generation sequencing and Array CGH technology.

**Results:**

We present the molecular findings for an Australian LCA cohort, sourced from the Australian Inherited Retinal Disease Registry & DNA Bank. *CEP290* and *GUCY2D* mutations, each represent 19% of unrelated LCA cases, followed by *NMNAT1* (12%). Genetic subtypes were consistent with other reports, and were resolved in 90% of this cohort.

**Conclusion:**

The high resolution rate achieved, equivalent to recent findings using whole exome/genome sequencing, reflects the progression from hypothesis (LCA Panel) to non‐hypothesis (RD Panel) testing and, coupled with Array CGH analysis, is a highly effective first‐tier test for LCA.

## Introduction

The clinical hallmark of Leber congenital amaurosis (LCA) is the onset of severe visual impairment at or soon after birth. These forms of infantile blindness represent the most severe inherited retinal dystrophies (IRDs) without major systemic features (den Hollander et al. 2008), and are characterized by severe visual handicap, nystagmus, sluggish pupils, and a severely subnormal or nondetectable electroretinogram (ERG) (Schappert‐Kimmijser et al. 1959). LCA represents ~5% of all IRDs (Schappert‐Kimmijser et al. 1959), with an estimated population frequency of between one in 30,000 (Koenekoop 2004) and one in 81,000 (Stone 2007) unrelated individuals.

LCA encompasses a group of heterogeneous disorders, with 24 genes currently implicated in pathogenesis (RetNet, 2017), leading to diverse clinical phenotypes posing challenges for diagnosis and patient management. Personalized therapies such as gene‐specific (gene augmentation), pharmacologic (oral retinoids/sodium 4‐phenylbutyrate [PBA]), or pharmacogenetic strategies (antisense oligonucleotides [AONs]/translational‐read‐through‐inducing drugs) (Bainbridge et al. 2008; Hauswirth et al. 2008; Maguire et al. 2008; Goldmann et al. 2010; Collin et al. 2012; Gerard et al. 2012; Koenekoop et al. 2014; Schwarz et al. 2015; Garanto et al. 2016; Li et al. 2016; Nagel‐Wolfrum et al. 2016; Parfitt et al. 2016) will be required to discern the most effective treatment (Chiang and Gorin 2016). Unraveling the genetic etiology of disease in individuals will thus be a prerequisite, and could identify potential candidates for personalized therapies, inform patient management (including diagnostic confirmation, dietary modifications, and reproductive options), and discriminate possible syndromic forms of disease.

Here, we present the molecular findings for an LCA cohort within the Australian population, sourced from the Australian Inherited Retinal Disease Registry (AIRDR) & DNA Bank. We genetically analyzed 45 affected and 82 unaffected individuals from 34 unrelated pedigrees to expedite impending clinical trials and to add to the knowledge of the genetic basis of IRDs. We report 39 mutations as a cause or a potential cause of LCA in 35 individuals, including 13 novel potentially disease‐causing variants.

## Materials and Methods

### Ethical compliance

Research was conducted in accordance with the Declaration of Helsinki, with informed consent provided. Ethics approval was granted by the Sir Charles Gairdner Hospital Human Research Ethics Committee (Human Ethics Approval Number 2001‐053).

#### Design

Fifty affected participants with a clinical diagnosis of LCA and 80 family members from 40 unrelated families were sourced from the AIRDR (De Roach et al. 2013). Demographic, pedigree, and clinical information was gathered by telephone, personal interview, completion of a questionnaire, and access to medical records, where available.

#### Mutational analysis

DNA samples were collected, processed and stored as detailed previously (De Roach et al. 2013). DNA was analyzed predominantly by targeted next‐generation sequencing (NGS) using disease‐specific SmartPanels (Chiang et al. 2015) and confirmed by Sanger sequencing. A progressive analysis was sometimes employed until the genetic cause was resolved. SmartPanel analysis, performed by the Casey Eye Institute (CEI) Molecular Diagnostics Laboratory (Portland, OR, USA), evolved throughout the study (gene information available on request). All exons and flanking intronic regions were sequenced, with nucleotide one corresponding to the A of the start codon ATG. Limited DNA was analyzed using LCA microarrays (versions 7‐9) performed by Asper Biotech Ltd (Tartu, Estonia), with targeted sequencing of variants performed by CEI, the Australian Genome Research Facility (AGRF) (Perth, WA, Australia), Asper Biotech, or Carver Laboratory (Iowa, USA). Partial gene deletions were identified by Array CGH performed by CEI (refer Table S1B). OMIM accession numbers/gene reference sequences utilized are; *AIPL1* (604393; NM_014336.3): *CEP290* (611755; NM_025114.3): *CRB1* (613835; NM_201253.2): *GUCY2D* (204000; NM_000180.3): *LCA5* (604537; NM_001122769.2): *NMNAT1* (608553; NM_022787.3): *RDH12* (612712; NM_152443.2): *RPE65* (204100; NM_000329.2): *RPGRIP1* (613826; NM_020366.3): *SPATA7* (604232; NM_018418.4): *TULP1* (613843; NM_003322.3).

#### Variant interpretation

Variant nomenclature is in accordance with Human Genome Variation Society recommendations (den Dunnen and Antonarakis 2000). Variants were scrutinized for pathogenicity utilizing a computer‐assisted method developed at the AIRDR (Huynh et al. 2016), including in silico modeling, interrogation of variation databases, scientific literature, and the clinical diagnosis, taking into account allele frequencies sourced from the ExAC Browser (Lek et al. 2016). Pathogenicity was interpreted in accordance with the American College of Medical Genetics guidelines (Richards et al. 2015). Pathogenicity related to disease segregation within the AIRDR database was not assessed due to insufficient informative meioses (Jarvik and Browning 2016); this information was sourced from publications. For in silico prediction, insertion/deletion variants were assessed using Mutation Taster (Schwarz et al. 2010), SIFT INDEL (Ng and Henikoff 2001), and VEST INDEL (Douville et al. 2016). Nonsense variants were assessed using Mutation Taster and, together with frameshift variants, were examined for predicted ability to invoke nonsense‐mediated decay (NMD). Missense variants were gauged using Mutation Taster, Align GVGD (Tavtigian et al. 2008), PolyPhen2 (Adzhubei et al. 2010), and SIFT. Splice site variants at consensus dinucleotides (±1/2) were automatically assigned a pathogenic status (Hellen 2009; Wallis et al. 2013) or assessed using Mutation Taster and Spliceman (Lim and Fairbrother 2012).

## Results

### AIRDR database information

**Figure 1:**
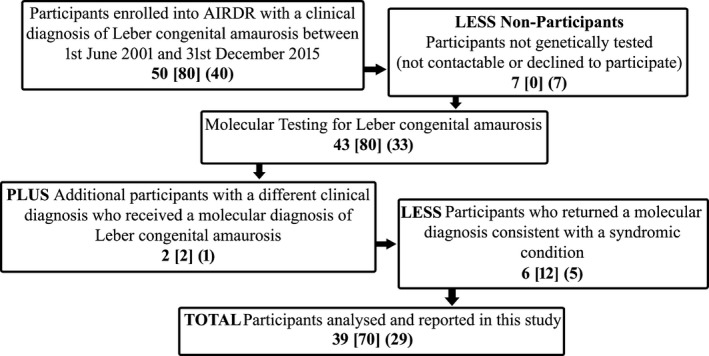
Flow diagram of the participants sourced from the AIRDR undertaking molecular testing who returned a molecular diagnosis of Leber congenital amaurosis, or had a clinical diagnosis of Leber congenital amaurosis but remained unresolved. Number of participants = number of affected individuals [number of unaffected individuals] (number of pedigrees).

Between 1 June 2001 and 31 December 2015, 50 individuals with a clinical diagnosis of LCA from 40 unrelated pedigrees were enrolled into the AIRDR from ophthalmologist referrals or directly through expressions of interest received from individuals. The cohort described herein includes all individuals contained within the registry with a clinical diagnosis of LCA and represents a subset of the Australian LCA population. In 2012, this cohort was considered sufficiently significant to be selected for analysis. At the time of the study, two individuals could not be contacted and five individuals declined to participate in genetic testing. DNA samples for the remaining 43 individuals and 80 unaffected family members from 33 pedigrees underwent molecular testing. An additional two siblings, affected at birth, from a consanguineous pedigree diagnosed with cone‐rod dystrophy or fundus albipunctatus returned a molecular diagnosis consistent with LCA and were added to the cohort and referred for clinical re‐evaluation. A further five individuals from four families returned a molecular diagnosis consistent with syndromic conditions (*CLN3;* Batten disease/*AHI1;* Joubert syndrome / *IFT140;* Mainzer‐Saldino syndrome) and were referred for clinical re‐evaluation. An additional individual (since deceased) had inconclusive results suggestive of a syndromic condition (*PEX1*; Infantile Refsum disease). These six individuals were excluded from further analysis. We hereby present the molecular findings for 39 affected and 70 unaffected individuals from 29 unrelated LCA pedigrees (Fig. 1).

### Molecular results

#### Variant detection

Targeted NGS SmartPanels and array analyses utilizing the DNA of 39 affected and 70 unaffected individuals from 29 unrelated pedigrees resolved the likely causative mutations for LCA in 26/29 (89.7%) pedigrees, with all individuals demonstrating autosomal recessive inheritance, and no sporadic cases identified. Considerable allelic heterogeneity was noted, with homozygosity occurring in only 4/26 (15.4%) families. Consanguinity was identified in only one family, indicating this was not a significant risk factor for disease in this cohort.

#### Pedigree information

Simplex cases represented 19/29 (65.5%) pedigrees and 10 pedigrees (34.5%) only had one other affected individual. Pedigree information detailing the genotype of affected individuals from 26 resolved pedigrees, categorized by gene function (detailed below; based on the format of Wright et al. 2010), is displayed in Table S1A. Biallelism and carrier status were determined by familial analysis of 63 unaffected individuals from 24/26 (92.3%) resolved pedigrees (Tables S1A/S1B).

#### Variant information and interpretation

A total of 41 variants were assessed for pathogenicity, including 39 disease‐causing or potentially disease‐causing variants and two benign variants. Variant information is detailed according to mutation type (Table S2), with additional information including NMD prediction and pathogenicity interpretation provided (Table S3).

#### Prevalence of mutations in LCA‐associated genes

**Figure 2:**
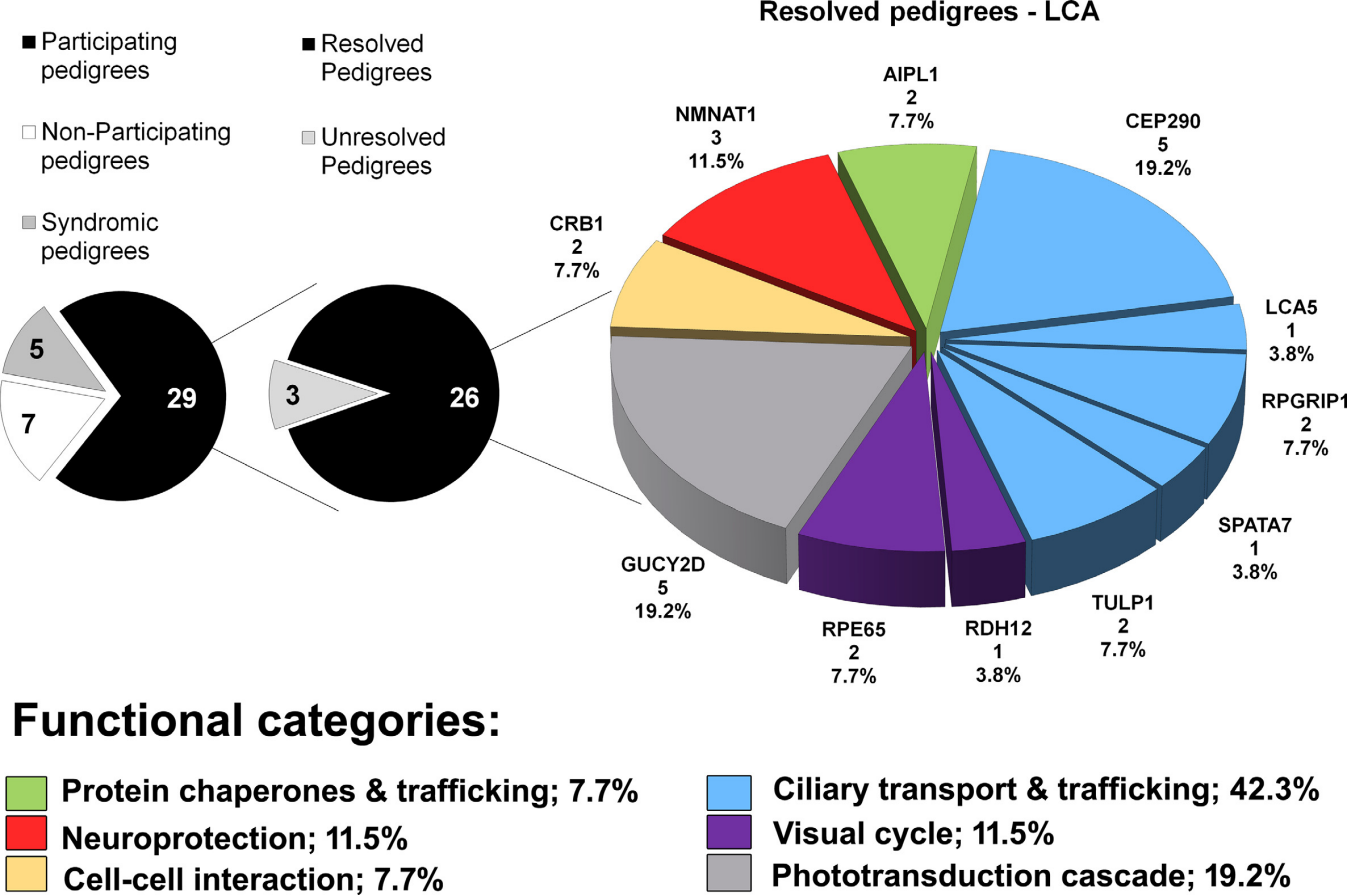
Diagram illustrating the prevalence of mutations in 11 genes associated with Leber congenital amaurosis within an Australian cohort. Labels show: **chart** ‐gene; number of pedigrees; prevalence: **legend** ‐ functional category; prevalence.

Variants considered likely candidates for disease in 26 resolved pedigrees occurred in 11 genes associated with diverse functions such as ciliary transport and trafficking, the visual cycle, phototransduction cascade, cell–cell interaction, neuroprotection, and protein chaperones and trafficking (Fig. 2).

#### Ciliary transport and trafficking

Variants in genes involved in this category represented 42.3% (11/26) of the cohort, with all pedigrees containing one or two variants resulting in protein truncation or NMD. The 16 variants detected included seven nonsense, six frameshifting, and three splice site‐related, and six were novel (Table 1/Table S1). This category was characterized by variants in five genes.

**Table 1:** Gene‐variant summary depicting pathogenic or likely pathogenic variants detected in resolved pedigrees

Gene	Total Variants detected	Total Affected	Total Pedigrees	Pedigrees with		Pedigrees with T/NMD			Variant subtype						Variant nature	
				Homoz variants	Comp Heteroz variants	0 allele	1 allele	2 alleles	Missense	Nonsense	Frame Shift Ins/Del	In‐frame Ins/Del	Splice Site	Intronic	Reported	Novel
Ciliary transport and trafficking – 42.3%																
Summary	**16**	**17**	**11**	**3**	**8**	**0**	**3**	**8**	**0**	**7**	**6**	**0**	**3**	**0**	**10**	**6**
*CEP290*	7	6	5	0	5	0	2	3	0	2	3	0	2	astop	5	2
*LCA5*	1	2	1	1	0	0	0	1	0	0	1	0	0	0	0	1
*RPGRIP1*	4	4	2	0	2	0	0	2	0	3	1	0	0	0	2	2
*SPATA7*	1	2	1	1	0	0	0	1	0	1	0	0	0	0	1	0
*TULP1*	3	3	2	1	1	0	1	1	0	1	1	0	1	0	2	1
Visual cycle – 11.5%																
Summary	**6**	**3**	**3**	**0**	**3**	**1**	**2**	**0**	**2**	**2**	**0**	**1**	**1**	**0**	**3**	**3**
*RDH12*	2	1	1	0	1^b^	0	1	0	1	1	0	0	0	0	2	0
*RPE65*	4	2	2	0	2^b^	1	1	0	1	1	0	1	1	0	1	3
Phototransduction cascade – 19.2%																
*GUCY2D*	7	5	5	1	4	1	4	0	3	1	3	0	0	0	6	1
Cell‐cell interaction – 7.7%																
*CRB1*	3	3	2	0	2	0	2	0	1	0	2	0	0	0	2	1
Neuroprotection – 11.5%																
*NMNAT1*	4	3	3	0	3	0	3	0	2	1	1	0	0	0	3	1
Protein chaperones and trafficking – 7.7%																
*AIPL1*	3	4	2	0	2	0	1	1	0	1	1	0	1	0	2	1
Total (*n*)	**39**	**35**	**26**	**4**	**22**	**2**	**15**	**9**	**8**	**12**	**13**	**1**	**5**	**0**	**26**	**13**
Total (%)				**15.4%**	**84.6%**	**7.7%**	**57.7%**	**34.6%**	**20.5%**	**30.8%**	**33.3%**	**2.6%**	**12.8%**	**0.0%**	**66.7%**	**33.3%**

Reference sequences: *AIPL1* NM_014336.3/*CEP290* NM_025114.3/*CRB1* NM_201253.2*/GUCY2D* NM_000180.3*/LCA5* NM_001122769.2/*NMNAT1* NM_022787.3/*RDH12* NM_152443.2*/RPE65* NM_000329.2*/RPGRIP1* NM_020366.3/*SPATA7* NM_018418.4*/TULP1* NM_003322.3.

a Intronic variant producing a nonsense mutation.

b Biallelism not determined; T/NMD = predicted truncating/nonsense‐mediated decay variants; Ins/Del = insertion/deletion. Summary information is denoted in bold.

#### *CEP290*

Of seven variants detected in five pedigrees (19.2%; 5/26), two were novel, five were predicted to invoke NMD, and two occurred at splice sites. There were four simplex pedigrees. All individuals possessed one or two NMD variants, with *CEP290:*c.2991+1655G>A identified in 4/5 pedigrees. This hypomorphic variant, well‐established as pathogenic for LCA, causes insertion of a cryptic exon and subsequent truncation (den Hollander et al. 2006). The final pedigree harbored a reported splice variant c.5587‐1G>C with the frameshifting c.3175dup. A large splicing change is predicted for the splice variant (Xiong et al. 2015), which may result in skipping of exon 41 (Perrault et al. 2007). Compound heterozygosity of c.1781T>A or c.3175dup with c.2991+1655G>A is reported for LCA (Yzer et al. 2012; Ellingford et al. 2016).

#### *LCA5*

A novel frameshifting insertion was detected homozygously in two affected individuals from one consanguineous pedigree (3.8%; 1/26). NMD is not predicted as the premature termination codon (PTC) (at c.1191) occurs <50 nucleotides from the 3′‐most exon‐exon junction (Nagy and Maquat 1998). The mutant protein is predicted to lose part of exon 7 and all of exon 8. There are four other reported examples of PTCs occurring after c.1191 (Boldt et al. 2011; Mackay et al. 2013; Wang et al. 2015) in the Human Gene Mutation Database (HGMD) (Stenson et al. 2014). The siblings were diagnosed with fundus albipunctatus and cone‐rod dystrophy, with the latter associated with light perception (LP) since birth, Franceschetti's oculodigital sign and keratoconus (Table S4). Mutations in *LCA5,* generally associated with LCA, have recently been reported to cause cone dystrophy (Chen et al. 2016). Due to the severity of the truncated variant and the clinical phenotype, the siblings were included here and referred for clinical re‐evaluation.

#### *RPGRIP1*

Of the four potentially disease‐causing variants detected in two pedigrees (7.7%; 2/26), all were predicted to cause NMD and two were novel. In one pedigree, both affected individuals possessed the c.1763‐8C>G and c.1219C>T variants *in cis*; the c.1219C>T variant should render the second variant irrelevant due to predicted NMD.

#### *SPATA7*

A homozygous variant, predicted to cause NMD, was detected in two affected individuals from one pedigree (3.8%; 1/26).

#### *TULP1*

Of the three variants detected in three affected individuals from two pedigrees (7.7%; 2/26), there was one novel frameshifting insertion, one nonsense mutation (both predicted to cause NMD), and one splice site change. The homozygous insertion c.524dup resulted from maternal isodisomy (detailed elsewhere; manuscript under preparation). Homozygosity for splice variant c.999+5G>C or nonsense variant c.1081C>T is pathogenic for early onset/juvenile RP (den Hollander et al. 2007; Ajmal et al. 2012) or LCA (Glockle et al. 2014; Guo et al. 2015), respectively.

#### Visual cycle

This category represented 11.5% (3/26) of the cohort, and included six variants, three of which were novel. There were two variants predicted to cause NMD, two missense mutations, one in‐frame deletion and one splice site change. All individuals represented simplex cases, with one pedigree containing no truncating or NMD variants and two pedigrees containing one NMD variant (Table 1/Table S1A). This category was characterized by variants in two genes.

#### *RDH12*

This genetic subtype consisted of one affected individual (3.8%; 1/26) who possessed one reported nonsense mutation expected to cause NMD and one reported missense mutation (Mackay et al. 2011). Biallelism was not determined due to lack of family DNA.

#### *RPE65*

A total of four variants in two affected probands were noted (7.7%; 2/26), three of which were novel. One individual possessed an in‐frame deletion and a nonsense mutation (NMD predicted). The second individual possessed a missense mutation and a splice site mutation (biallelism not determined due to lack of family DNA), together with a reported mutation in *RPGRIP1*, which may act as a disease modifier (Zernant et al. 2005).

The remaining categories are each represented by variants within one gene.

#### Phototransduction cascade

Variants within *GUCY2D* represented 19.2% (5/26) of the cohort and all 5 pedigrees involved simplex cases. Of seven variants, three were missense, one was nonsense and three were frameshifting. Of the five pedigrees, four contained one truncating/NMD variant and one contained no truncating/NMD variants. *GUCY2D* missense mutations constituted 37.5% of all missense mutations detected in this study.

The reported c.307G>A (E103K) missense variant (Stone 2003, 2007; Henderson et al. 2007; Borman 2015) was detected in two pedigrees, with either c.91dup or c.2595del, both reported for LCA (Hanein et al. 2004; Jacobson et al. 2013; Ellingford et al. 2016). While functional studies have not been conducted, the E103K substitution occurs within the extracellular ligand‐binding domain, and the similar c.308A>T (E103V) variant, pathogenic for LCA (Li et al. 2011), adds weight to the evidence for pathogenicity due to changes at this conserved position.

The c.2302C>T missense variant (Stone 2007) was discovered in two pedigrees, one homozygously, and one in combination with c.2516del. This variant is well‐established as pathogenic for LCA (Hanein et al. 2004; Yzer et al. 2006; Simonelli et al. 2007; Coppieters et al. 2010, 2012; Li et al. 2011). Peshenko et al. (2010) demonstrate altered docking of GCAP1 on GUCY2D in vitro due to this variant. Zulliger et al. (2015) showed that the RD3/GUCY2D/GCAP1 complex is affected in R768W mutant mice, and in transfected COS‐7 cells it results in altered GUCY2D trafficking to the outer segments. The pedigree homozygous for c.2302C>T represents one of only two pedigrees not harboring truncating or NMD variants. The remaining pedigree had one missense c.2383C>T variant (Pasadhika et al. 2010; Roman et al. 2015) and one novel nonsense variant c.2646C>G, predicted to induce NMD.

#### Cell–cell interaction

*CRB1* mutations were detected in three affected individuals from two pedigrees (7.7%; 2/26), including one simplex case, and comprised three variants, including one novel variant. The c.2843G>A missense mutation was compound heterozygous in both pedigrees, with the remaining frameshift variants predicted to cause NMD. Compound c.[2843G>A];[613_619del] heterozygotes have been described for LCA (Corton et al. 2013), with c.2843G>A occurring in 23–31% of total *CRB1* alleles (Hanein et al. 2004; Corton et al. 2013). Interrogation of the AIRDR database identified this variant in three autosomal recessive retinitis pigmentosa pedigrees.

#### Neuroprotection

*NMNAT1* mutations were detected in three simplex pedigrees (11.5%; 3/26). Of four variants detected (including one novel variant), two were missense and two were predicted to truncate the protein; c.364del is predicted to remove the last part of exon 4 and all of exon 5, and c.507G>A lacks the last 110 amino acids but may retain some residual catalytic activity (Perrault et al. 2012). Chiang et al. (2012) detected this variant in combination with c.769G>A (as per pedigree 1565) in 4/11 cases of LCA with *NMNAT1* mutations. Two additional *NMNAT1* mutations causing PTCs without NMD are reported for LCA.

The c.364del and c.769G>A variants each occurred in two pedigrees, with one pedigree sharing these two variants, as reported for LCA (Perrault et al. 2012). The c.364del variant in both pedigrees was inherited from fathers of Maltese descent. The c.769G>A variant, occurring in >70% of LCA9 cases (Sasaki et al. 2015), was detailed previously (Chiang et al. 2012; Falk et al. 2012; Koenekoop et al. 2012; Perrault et al. 2012; Siemiatkowska et al. 2014b; Bravo‐Gil et al. 2016). Using in vitro and in vivo functional assays, Koenekoop et al. (2012) and Sasaki et al. (2015) agree this mutation could render the enzyme susceptible to stress‐induced changes in protein folding and enzyme activity. Siemiatkowska et al. (2014a) deduced this as a hypomorphic variant almost always causing LCA in combination with more severe *NMNAT1* variants, consistent with our findings.

#### Protein chaperones and trafficking

*AIPL1* variants were detected in four affected individuals from two pedigrees (7.7%; 2/26). Three variants were detected, including one nonsense, one novel frameshifting deletion, and one splice site variant. The c.834G>A nonsense variant (Testa et al. 2011; Aboshiha et al. 2015) produces a truncated protein lacking the C‐terminal 107 amino acids, including most of the TPR3 domain, disrupting AIPL1‐NUB1 binding and forming cytoplasmic inclusions (van der Spuy et al. 2002; Kanaya et al. 2004). Sohocki et al. (2000) detected the c.[834G>A];[c.277‐2A>G] combination in one LCA individual of French ethnicity. Splice site assays indicate that the c.277‐2A>G variant abolishes the intron two splice acceptor site and deletes exon 3, and may alter the transcript profile in this tissue (Bellingham et al. 2015).

#### Unresolved pedigrees

At the time of analysis, the genetic cause of disease in three pedigrees remained unresolved (10.3%; 3/29).

#### Variant subtypes

In total, 39 disease‐causing or potentially disease‐causing variants were detected in 35 affected individuals from 26 pedigrees (Table 1), including 13 novel variants (33.3%). Of these 39 variants, 13 (33.3%) were frameshifting, 12 (30.8%) were nonsense and eight (20.5%) were missense variants. Only five variants (12.8%) were detected in splicing regions, and one variant (2.6%) harbored an in‐frame deletion. The variant containing the intronic nucleotide substitution was categorized as a nonsense mutation due to the truncating effect (*CEP290:*c.2991+1655G>A).

In two pedigrees (7.7%; *RPE65* and *GUCY2D*), no variants that were predicted to result in protein truncation or NMD were detected. A further 15 pedigrees (57.7%) harbored one such allele, and nine pedigrees (34.6%) harbored two such alleles. Of the 12 nonsense variants, 10 are predicted to be recognized by the NMD machinery. Similarly, of the 13 frameshifting variants, 11 are predicted to invoke NMD.

### Phenotypic information

#### Comparison of available clinical or participant‐derived information

Available clinical information was sourced from ophthalmic reports and from participant‐derived information provided in questionnaires and telephone interviews. Information regarding clinical signs and symptoms characteristic of LCA (Table S4) were summarized and displayed by functional category/gene (Table 2). Nystagmus, severe visual impairment, markedly abnormal ERG, anterior segment abnormalities, and fundus changes were reported characteristics.

**Table 2:** Comparison of available clinical or participant‐derived information summarized by functional category/gene

Summary										
Gene	# Affecteds	# Pedigrees	Nystagmus	Visual Acuity (range)	ERG/VEP	Photophobia	Nyctalopia	Ref Err	Ant Seg Abn	Retinal fundus changes /comments
Ciliary transport and trafficking										
*CEP290*	6	5	✓	LP‐NLP since birth	Bilaterally flat ERG tracings (3) No data (3)	✓ (1)			K(1) ×(2)	Not drawn to bright lights (1) Pigmentary changes (1) Attenuated retinal vessels (1) Eye poking (3) Fundi normal at 0^7^ (1) No fundi data (3)
*LCA5*	2	1	✓	LP‐NLP since birth	Bilaterally flat ERG tracings (2)	✓ (1)	× (1)	× (1)	K+C (1)	Eye poking (1) Dense asteroid hyalosis^OD^; Waxy‐looking optic disc^OS^; attenuated retinal vessels; bone spicule pigment clumping (very marked parafoveally); generalized retinal thinning; congenital Descemet's membrane abnormalities; All changes in sibling with LP [no fundus data for other sibling] Sibling (NLP) was previously always drawn to light/experienced flashing lights/floating spots (1)
*RPGRIP1*	4	2	✓	≤6/60	Bilaterally flat ERG tracings (2) No data (2)	✓ (2)	✓ (2)	H (1)	× (4)	Constricted fields of vision (1) Can see sparks of gold/bright light continuously if concentrating (1) Pigmentary changes (1) No fundi data (3)
*SPATA7*	2	1	✓	LP at birth to LB in second decade	No data	× (2)	× (2)			Corneal transplant at 27 improved vision (1) Siblings both homozygous for same variant but phenotypically variable – 1 LB in second decade/1 LP only from birth
*TULP1*	3	2	✓	≤6/60	No data (3)	✓ (1) × (2)	✓ (3)	M (1)		Notable deterioration in vision in early‐mid second decade (1) Eye poking (2) Stares at bright light (2) Central vision blurred (3) A little color blind (1) No fundi data (3)
Visual cycle										
*RDH12*	1	1	✓	LP	No data	× (1)	✓ (1)			Rods and cones not developed properly, RPE pigment changes; strabismus and nyctalopia from birth; color vision deteriorated until absent at 13; vision stable until early 20s; progression until 38 to LP in sunshine, then blackness/like looking through fog
*RPE65*	2	2	✓	≤6/60	Bilaterally flat ERG tracings (1) No data (1)	× (2)	✓ (2)		× (1)	Pale optic nerve heads/prominent optic nerve sheaths (1) Prefers bright light (1) Upbeat nystagmus (1)
Phototransduction cascade										
*GUCY2D*	5	5	✓	LP since birth	Bilaterally flat ERG tracings (3) Severely reduced bilaterally at 0^6^ (1)	× (2)		HH (1) M (1)	× (3)	Eye poking (3) Drawn to bright light (3) Faintly present direct pupillary responses (1) Sluggish pupillary responses (2) Fundi appear normal (4) / No fundi data (1)
Cell–cell interaction										
*CRB1*	3	2	✓	3/60 ‐ LP	Bilaterally flat ERG tracings (2) No data (1)	✓ (1)	✓ (3)		C (1) × (2)	Gradual disease progression until notable deterioration at 15–17 (2), with stability then further deterioration at 23 (1) Another noticed deterioration at 30 (1) Pigmentary retinopathy (2) Sluggish pupillary responses (2) Color vision absent (2) Paramacular donut ring of vision (1) Small gliotic discs, atrophic macula, arterioles attenuated and constricted fields (1) Previous flashing lights/floating spots (1)
Neuroprotection										
*NMNAT1*	3	3	✓	LP‐NLP since birth	No data	✓ (3)				No fundi data (3)
Protein chaperones and trafficking										
*AIPL1*	4	2	✓	LP since birth	No data	× (3)	× (3)		K (1) × (3)	Eye poking (4) / Enophthalmia (1) Not drawn to lights (1) Stared at bright lights when younger (1) Flashing lights in vision before NLP (1) No fundi data (4)

✓ = present, × = not present, blank field = no data available; numbers in brackets represent number of individuals with or without trait; age reported = years^months^; Ant Seg Abn, anterior segment abnormalities; C, cataract/s; ERG, electroretinogram; H, hypermetropia; HH, high hypermetropia; K, keratoconus; LB, legally blind; LP, light perception; M, myopia; NLP, no light perception; OD, oculus dexter (right eye) OS, oculus sinister (left eye); Ref Err, refractive errors; RPE, retinal pigment epithelium; VEP, vision evoked potentials.

### Clinical signs and symptoms

#### Nystagmus

Nystagmus was reported for all individuals with available data.

#### Visual acuity

The most severe visual deficiency was noted in individuals with *AIPL1, CEP290, GUCY2D, LCA5,* and *NMNAT1* mutations, who had either LP or no light perception (NLP) at birth. More variable deficiency was noted in three individuals with *CRB1* mutations (two reported LP; one had a visual acuity of 3/60; variable between siblings). Similarly, of the two siblings with a homozygous *SPATA7* mutation, one had LP since birth, and the other has been legally blind (LB) since their second decade. These data demonstrate intrafamilial phenotypic variability despite harboring the same causative variants. All three individuals with *CRB1* mutations reported progressive visual deterioration, as did the individual with *RDH12* mutations who progressed to LP at 38 years of age. Less severe visual deficiency (≤6/60) was noted in the remaining pedigrees (*RPE65, RPGRIP1, TULP1*).

#### Electroretinography

Consistent with the diagnosis of LCA, bilaterally flat ERG tracings were noted for all reported individuals with *CEP290, CRB1, GUCY2D, LCA5, RPE65,* and *RPGRIP1* genotypes. No ERG data were available for *AIPL1, NMNAT1, RDH12, SPATA7,* or *TULP1* genotypes.

#### Photophobia

Photophobia was reported for nine individuals, including *NMNAT1* (all three individuals), *RPGRIP1* (two individuals from one pedigree), *LCA5* (one individual with LP at birth), and *CEP290*,* CRB1,* or *TULP1* (one individual each) genotypes.

#### Nyctalopia

Symptoms of night blindness were reported by 11 individuals, including *CRB1* or *TULP1* mutations (all individuals; three each group), *RPGRIP1* or *RPE65* mutations (two individuals each) and *RDH12* mutations (one individual).

#### Light‐gazing

Individuals with *AIPL1*,* GUCY2D*,* LCA5,* and *TULP1* mutations reported attraction to light. Some individuals with *AIPL1* and *CEP290* mutations reported no attraction to light.

#### Refractive errors

Either high hypermetropia or myopia was reported by two individuals with *GUCY2D* mutations. An individual with *RPGRIP1* mutations reported hypermetropia and one *TULP1*‐affected individual reported myopia.

#### Anterior segment abnormalities

Cataract was reported in two individuals (*CRB1, LCA5*) and keratoconus was identified in three individuals (*AIPL1, CEP290, LCA5*), of which two reported eye poking.

#### Franceschetti's oculodigital sign

Eye poking was reported in 13 individuals: *AIPL1* (4/4), *CEP290* (3/5), *GUCY2D* (3/5), *LCA5* (1/2), and *TULP1* (2/3). Of the two siblings with homozygous *LCA5* mutation, only one sibling displayed eye poking. Eye poking was not reported for individuals with mutations in *CRB1, NMNAT1, RDH12, RPE65, RPGRIP1,* or *SPATA7*.

#### Fundus changes

Pigmentary changes were documented in individuals with mutations in *CEP290, CRB1, LCA5, RDH12,* and *RPGRIP1*. Attenuated retinal vessels were reported in individuals with *CEP290, CRB1,* and *LCA5* mutations. An individual with an *LCA5* mutation had dense asteroid hyalosis and a waxy‐looking optic disc, while one individual with *RPE65* mutations exhibited pale optic nerve heads and prominent optic nerve sheaths. *CRB1* mutation in one pedigree resulted in small gliotic discs and atrophic macula in one individual and a paramacular donut ring of vision in the other. In stark contrast, the fundi of individuals with *GUCY2D* mutations appeared normal.

#### Summary by functional category/gene

In summation, subtly different clinical manifestations were identified within the different genetic subtypes, which have been summarized and displayed by functional category/gene (Table 2).

## Discussion

In this study, 39 affected and 70 unaffected participants from 29 unrelated pedigrees were molecularly targeted for variants in LCA‐ or IRD‐related genes. All affected individuals inherited this condition in an autosomal recessive manner, with no sporadic cases detected. At the time of the study, this LCA cohort comprised 1.4% of affected individuals within the AIRDR.

The use of NGS with copy number variation detection increases the efficiency of variant detection in LCA (Eisenberger et al. 2013). Using predominantly NGS coupled with Array CGH, we resolved the cause or potential cause of primary disease in 90% of LCA pedigrees within this cohort, implicating 11 genes known to be associated with LCA. Biallelism was demonstrated in >90% of resolved cases.

The 90% resolution achieved in this study is comparable to recent studies for LCA using targeted NGS panels (80%; Bernardis et al. 2016) or whole exome/genome sequencing (89%; it is acknowledged that this study was enriched for intractable cases; Carss et al. 2017). This is attributable to the progression from hypothesis (LCA panel) to non‐hypothesis (RD Panel) testing (refer Table S1B), which provided an alternative molecular diagnosis in 19% (6/31) pedigrees. We now routinely utilize RD Panels when investigating most IRDs to abrogate the impact of phenotypic heterogeneity on the clinical diagnosis.

In this Australian population, 66% of pedigrees contained simplex cases, with a further 34% involving one other affected sibling, hence the low number of informative meioses. In total, 39 disease‐causing or potentially disease‐causing variants were established, including 13 novel variants. Considerable allelic heterogeneity was demonstrated, with homozygosity detected in only four pedigrees, indicating consanguinity was not a significant risk factor. Overall, variants were predominantly nonsense or frameshift, and >50% are predicted or shown to be recognized by the NMD machinery. With the exception of two pedigrees, all pedigrees contained either one or two severe variants resulting in PTC or NMD, consistent with literature relating variant severity to the severity of these diseases (Wang et al. 2009). *GUCY2D* variants were the exception, where 43% of variants were missense substitutions. The higher rate of missense mutations noted here for *GUCY2D* is in keeping with previous reports for LCA (Hanein et al. 2004).

Genes involved in ciliary transport and trafficking were responsible for disease in 42% of this cohort, mostly attributable to *CEP290* which, like *GUCY2D* (phototransduction cascade), was disease‐causing in 19% of the cohort. Similar to previous reports (den Hollander et al. 2008), *CEP290* and *GUCY2D* were the most predominant causes of LCA, representing over one‐third of this group. At the time of analysis, three pedigrees (10%) remain unresolved, with *NMNAT1* (identified in three pedigrees), *AIPL1, CRB1, RPE65, RPGRIP1, TULP1, LCA5, RDH12,* and *SPATA7* accounting for the remaining pedigrees.

The most common variant detected was *CEP290:*c.2991+1655G>A, consistent with its determination as a founder mutation (den Hollander et al. 2006; Perrault et al. 2007). This hypomorphic variant produces a small amount of correctly spliced product. It has been suggested that LCA patients with *CEP290* mutations have a small amount of residual CEP290 activity, in contrast to the complete loss of function observed in Joubert syndrome (den Hollander et al. 2006), although this has been debated (Perrault et al. 2007). This general observation is borne out in this study, with 3/4 individuals with the *CEP290:*c.2991+1655G>A variant harboring a second variant predicted to cause NMD. As a frequent mutation associated with LCA, the use of AONs to almost fully restore normal splicing and protein synthesis in vitro in tissue of LCA‐affected individuals is promising (Collin et al. 2012; Gerard et al. 2012; Garanto et al. 2016; Parfitt et al. 2016). In contrast, *GUCY2D* variants were noticeably more diverse, with only two variants present in more than one pedigree.

From a clinical perspective, ophthalmic investigation identified characteristic signs and symptoms of LCA. Some phenotypic variability in visual acuity, anterior segment abnormalities, fundus changes, and the presence or absence of associated ocular signs and symptoms was noted between genetic subtypes. Individuals with *AIPL1, CEP290, GUCY2D, LCA5,* and *NMNAT1* mutations exhibited the most severe visual dysfunction (LP or NLP at birth). Due to less severe visual dysfunction, individuals with *RPGRIP1* mutations showed greater variation in severity and, consistent with our data, *RPE65* mutations result in less severe visual dysfunction (Simonelli et al. 2007; Pasadhika et al. 2010). Pedigrees with *SPATA7* and *CRB1* mutations showed intrafamilial variability in visual dysfunction despite homozygosity for the same causative variants; variability in visual acuity for *CRB1*‐affected individuals has been documented (Simonelli et al. 2007). For some individuals, fundus examination indicated pigmentary changes, vascular attenuation, optic disc changes and maculopathy. In contrast to other genotypes, the fundi of individuals with *GUCY2D* mutations appeared normal, consistent with previous reports (Pasadhika et al. 2010; Jacobson et al. 2013).

Emerging precision strategies promise great hope for ameliorating visual handicap in individuals afflicted with IRDs. For instance, subretinal *RPE65* gene‐specific therapy in LCA patients (ages 9–45) appears to promote remyelination of visual pathways resulting in enhanced visual function (Ashtari et al. 2015). In contrast, LCA patients with *AIPL1* mutations would benefit from intervention within the first few years (Aboshiha et al. 2015). Pharmacogenetic (AONs) and pharmacologic (oral retinoids, PBA) intervention has also proven beneficial (Collin et al. 2012; Koenekoop et al. 2014; Li et al. 2016; Parfitt et al. 2016) and combined therapies may prove optimum (Li et al. 2016). Collectively, this indicates a variable window for optimal treatment for different LCA‐subtypes and strategies, with the general consensus being that earlier intervention will be optimal (Maguire et al. 2009; Weleber et al. 2016). Discovery of the causative genes is thus paramount to successful patient management.

The severe and congenital nature of LCA places these IRDs at the forefront for identifying causative mutations and exploring personalized therapies. Due to the rare and highly variable nature of these diseases, an international pool of recruits may be required to promote research into personalized therapies (Estrada‐Cuzcano et al. 2012; Aboshiha et al. 2015). Conducting global genetic analyses parallel with research into gene‐specific, pharmacogenetic, or pharmacologic therapy will be a key determinant in the timely deliverance of future treatment therapies.

Finally, we cannot explain the discrepancy between our representation of LCA constituting 1.4% of all IRDs in our database and the expected rate of ~5%. It is feasible that pediatric ophthalmologists or clinical geneticists may not be aware of the AIRDR and thus do not refer relevant families for enrolment. Alternatively, it may be that some individuals with LCA do not maintain contact with their clinicians due to lack of treatment options. Given the promise of future therapies, these individuals should be encouraged to have ongoing follow‐up.

In conclusion, the use of targeted NGS SmartPanels coupled with Array CGH analysis is a highly effective first‐tier test for LCA. Using these tools together with clinical‐ and patient‐derived data, we revealed considerable allelic and phenotypic heterogeneity in individuals harboring predominantly severe variants in 11 genes associated with LCA. We identified the likely causative variants in 90% of LCA pedigrees within this Australian cohort, provided an alternative molecular diagnosis for 19% of pedigrees, and contributed to the understanding of the genetic basis of LCA.

## Conflict of Interest

No authors of this paper have any competing interests related to this research.

## Supporting information


**Table S1.** (A) Pedigree information displayed by functional categories of genes, identifying genetic variants that account for or are likely to account for disease in this cohort.

**Table S1.** (B) Pedigree information displayed by functional categories of genes, identifying progressive genetic testing methodologies employed in this study.

**Table S2.** Pathogenicity data for genetic variants that account for or are likely to account for disease in this cohort, displayed by variant subtype.

**Table S3.** Variant information and interpretation.

**Table S4.** Comparison of available clinical or participant‐derived information displayed by functional category.
