# Supplementary material for: The genetic profile of Leber congenital amaurosis in an Australian cohort
Source: Mol Genet Genomic Med. 2017 Aug 22;5(6):652–67. doi: 10.1002/mgg3.321 (PMC5702575; doi:10.1002/mgg3.321)
Supplement: Supplementary file 1 — Table S1. (A) Pedigree information displayed by functional categories of genes, identifying genetic variants that account for or are likely to account for disease in this cohort. [file MGG3-5-652-s001.pdf]

Supplementary Table 1A: Pedigree information displayed by functional categories of genes, identifying genetic variants that account for or are likely to account for disease in this cohort.

Family ID	Proband Variant ID Nucleotide changes		Siblings		Other family members	Comments
	Paternal Allele	Maternal Allele	Allele 1	Allele 2		
**Ciliary transport and trafficking**						
***CEP290* (NM_025114.3)**						
0623	c.4625_4626insCATG	c.2991+1655A>G	WT	WT	-	
**1015**	c.2991+1655A>G	c.3181_3182del	c.3181_3182del	WT	-	-
**1212**	c.2991+1655A>G	c.1781T>A	WT	WT	-	
**1576**	c.5587-1G>C	c.3175dup	c.3175dup	WT	-	-
			c.3175dup	WT		
			c.5587-1G>C	WT		
1950	c.2991+1655A>G	c.297+3A>G	c.2991+1655A>G	WT	2nd cousins (maternal)	
	c.2991+1655A>G	c.297+3A>G	-	-		
***LCA5* (NM_001122769.2)**						
1894	c.1144_1147dup	c.1144_1147dup	c.1144_1147dup	c.1144_1147dup	-	**Consanguinity** Clinical diagnosis of fundus albipunctatus (1) and cone-rod dystrophy (1)
***RPGRIP1* (NM_020366.3)**						
0479	c.1219C>T;1763-8C>G	c.exon19del chr14:g.(21798302_21798377)_(21798551_21799045)del (hg19,NC000014.8)	c.1219C>T; 1763-8C>G	c.exon19del		
1642	c.2935C>T	c.1447C>T	c.1447C>T	c.2935C>T	-	+RPGRIP1 c.1639G>T (benign)
***SPATA7* (NM_018418.4)**						
1543	c.763C>T	c.763C>T	c.763C>T	c.763C>T	-	-
			c.763C>T	WT		
			WT	WT		

TULP1 **(NM_003322.3)**						
1620	WT	c.524dup<br>c.524dup	WT	WT	Carrier c.524dup (maternal first cousin once removed)	Maternal isodisomy of chromosome 6; also harbours a variant in *RP1* (c.1267-1656delinsA p.Ala423Asnfs*11)
2175	c.1081C>T	c.999+5G>C	c.999+5G>C	c.1081C>T	-	-
**Visual cycle**						
RDH12 **(NM_152443.2)**						
**1425**	c.316C>T[NT] and c.697G>C[NT]		DNWTP		None	Biallelism not determined
RPE65 **(NM_000329.2)**						
**1404**	c.951_956del	c.130C>T	-	-	-	
**1723**	c.1040G>C[NT] and c.726-1G>A[NT]		-	-	Aboriginal family	Biallelism not determined<br>+ RPGRIP1 c.1767G>T p.Gln589His
**Phototransduction cascade**						
GUCY2D **(NM_000180.3)**						
**0535**	c.307G>A	c.2595del	-	-	-	
**1836**	c.2302C>T	c.2516del	-	-	-	-
**2019**	c.91dup	c.307G>A	-	-	-	-
**2123**	c.2646C>G	c.2383C>T	-	-		+ (paternal) interstitial deletion 2q13
**2599**	c.2302C>T	c.2302C>T	-	-		+ GUCY2D c.2345T>A (benign)

**Cell – cell interaction**						
***CRB1* (NM_201253.2)**						
0306	c.1793del	c.2843G>A	c.1793del	c.2843G>A	-	-
			c.1793del	WT		
**2274**	c.2843G>A	c.613_619del[BD]	c.613_619del	WT	-	Maternal DNA not available; allele identified in sibling
**Neuroprotection**						
***NMNAT1* (NM_022787.3)**						
**1565**	c.769G>A	c.507G>A	-	-	-	-
**1819**	c.500A>G	c.364del	-	-	-	Paternal c.364delA Maltese descent
**1965**	c.364del	c.769G>A	c.364del	WT	-	Paternal c.364delA Maltese descent
**Protein chaperones and trafficking**						
***AIPL1* (NM_014336.3)**						
2030	c.356_359del[BD]	c.834G>A	c.356_359del	c.834G>A		Paternal DNA not available; allele identified in sibling
			c.356_359del	WT		
			WT	WT		
2272	c.834G>A[BD]	c.277-2A>G	c.834G>A	c.277-2A>G	Carrier c.834G>A (paternal great aunt)	Paternal DNA not available; allele identified in paternal family

Pedigree ID in Bold indicates simplex case. BD = by default (parental DNA not available but variant detected in related family member); DNWTP = did not wish to participate; NT = not tested; WT = wildtype alelle. All heterozygous and homozygous WT individuals were unaffected.
