# Supplementary material for: The genetic profile of Leber congenital amaurosis in an Australian cohort
Source: Mol Genet Genomic Med. 2017 Aug 22;5(6):652–67. doi: 10.1002/mgg3.321 (PMC5702575; doi:10.1002/mgg3.321)
Supplement: Supplementary file 2 — Table S1. (B) Pedigree information displayed by functional categories of genes, identifying progressive genetic testing methodologies employed in this study. [file MGG3-5-652-s002.docx]

**Supplementary Table 1B: Pedigree information displayed by functional categories of genes, identifying progressive genetic testing methodologies employed in this study.**

| **Family ID** | **Proband Variant ID**  **Nucleotide changes** | | **Siblings** | | **Testing methodology** | **Date of analysis** |
| --- | --- | --- | --- | --- | --- | --- |
|  | **Paternal Allele** | **Maternal Allele** | **Allele 1** | **Allele 2** |  |  |
| **Ciliary transport and trafficking** | | | | | | |
| ***CEP290* (NM_025114.3)** | | | | | | |
| 0623 | c.4625_4626insCATG | c.2991+1655A>G | WT | WT | Asper LCA Array  **CEI LCA Panel – 19 genes** | -  **30/09/2014** |
| **1015** | c.2991+1655A>G | c.3181_3182del | c.3181_3182del | WT | Asper LCA Array  **CEI LCA Panel – 19 genes** | -  **30/09/2014** |
| **1212** | c.2991+1655A>G | c.1781T>A | WT | WT | Asper LCA Array  **CEI LCA Panel – 19 genes** | -  **30/09/2014** |
| **1576** | c.5587-1G>C | c.3175dup | c.3175dup | WT | Asper LCA Array  AGRF RPE65 sequencing  AGRF AIPL1 sequencing  **CEI LCA Panel – 22 genes** | **-**  **-**  **-**  **30/09/2014** |
|  |  |  | c.3175dup | WT |  |  |
|  |  |  | c.5587-1G>C | WT |  |  |
| 1950 | c.2991+1655A>G | c.297+3A>G | c.2991+1655A>G | WT | **CEI LCA Panel – 19 genes** | **30/09/2014** |
|  | c.2991+1655A>G | c.297+3A>G | - | - |  |  |
| ***LCA5* (NM_001122769.2)** | | | | | | |
| 1894 | c.1144_1147dup | c.1144_1147dup | c.1144_1147dup | c.1144_1147dup | CEI STGD/Mac Dys panel v1  **CEI RD Panel v4**  **CEI RD Panel v8** | 13/02/2014  **02/02/2015**  **03/08/2015** |
| ***RPGRIP1* (NM_020366.3)** | | | | | | |
| 0479 | c.1219C>T;1763-8C>G | c.exon 19 del  chr14:g.(21798302_21798377)_(21798551_21799045)del (hg19,NC000014.8) | c.1219C>T;  1763-8C>G | c.exon19del | Asper LCA Array  AGRF RPE65 sequencing  AGRF AIPL1 sequencing  **CEI RD Panel v8**  **CEI Array CGH** | -  23/01/2013  23/07/2013  **03/08/2015**  **03/08/2015** |
| 1642 | c.2935C>T | c.1447C>T | c.1447C>T | c.2935C>T | Asper LCA Array  AGRF RPE65 sequencing  AGRF AIPL1 sequencing  **CEI RD Panel v8** | 05/10/2012  23/01/2013  23/07/2013  **31/07/2015** |
| ***SPATA7* (NM_018418.4)** | | | | | | |
| 1543 | c.763C>T | c.763C>T | c.763C>T | c.763C>T | Asper LCA Array  AGRF RPE65 sequencing  AGRF AIPL1 sequencing  **CEI RD Panel v8** | 12/08/2013  12/08/2013  12/08/2013  **03/08/2015** |
|  |  |  | c.763C>T | WT |  |  |
|  |  |  | WT | WT |  |  |
| ***TULP1* (NM_003322.3)** | | | | | | |
| 1620 | WT | c.524dup  c.524dup | WT | WT | Asper LCA Array  AGRF RPE65 sequencing  AGRF AIPL1 sequencing  Asper ARRP Array  **CEI RD Panel v9** | 17/08/2012  23/01/2013  12/08/2013  24/06/2013  **16/06/2015** |
| 2175 | c.1081C>T | c.999+5G>C | c.999+5G>C | c.1081C>T | **CEI LCA Panel - 22 genes** | **10/03/2015** |
| **Visual cycle** | | | | | | |
| ***RDH12* (NM_152443.2)** | | | | | | |
| **1425** | c.316C>T^NT^ and c.697G>C^NT^ | | DNWTP | | **CEI RD Panel v8** | **03/08/2015** |
| ***RPE65* (NM_000329.2)** | | | | | | |
| **1404** | c.951_956del | c.130C>T | - | - | Asper LCA Array  **AGRF RPE65 sequencing**  **Carver Targeted sequencing** | 05/10/2012  **05/06/2013**  **26/06/2014** |
| **1723** | c.1040G>C^NT^ and c.726-1G>A^NT^ | | - | - | **CEI LCA Panel - 22 genes** | **27/11/2014** |
| **Phototransduction cascade** | | | | | | |
| ***GUCY2D* (NM_000180.3)** | | | | | | |
| **0535** | c.307G>A | c.2595del | - | - | Asper LCA Array  **CEI LCA Panel - 19 genes** | -  **30/09/2014** |
| **1836** | c.2302C>T | c.2516del | - | - | AGRF RPE65 sequencing  AGRF AIPL1 sequencing  **Asper LCA Array** | 12/08/2013  12/08/2013  **12/08/2013** |
| **2019** | c.91dup | c.307G>A | - | - | **CEI LCA Panel – 19 genes** | **02/10/2014** |
| **2123** | c.2646C>G | c.2383C>T | - | - | **Sanger sequencing (external)**  **Array CGH (external)**  **AGRF Targeted sequencing** | **-**  **-**  **17/05/2016** |
| **2599** | c.2302C>T | c.2302C>T | - | - | **Carver LCA Array**  **AGRF Targeted sequencing** | **^-^**  **17/05/2016** |
| **Cell – cell interaction** | | | | | | |
| ***CRB1* (NM_201253.2)** | | | | | | |
| 0306 | c.1793del | c.2843G>A | c.1793del | c.2843G>A | Asper LCA Array  **CEI LCA Panel – 19 genes** | -  **30/09/2014** |
|  |  |  | c.1793del | WT |  |  |
| **2274** | c.2843G>A | c.613_619del^BD^ | c.613_619del | WT | **CEI RD Panel v8** | **17/08/2015** |
| **Neuroprotection** | | | | | | |
| ***NMNAT1* (NM_022787.3)** | | | | | | |
| **1565** | c.769G>A | c.507G>A | - | **-** | Asper LCA Array  AGRF RPE65 sequencing  **CEI LCA Panel – 19 genes** | 17/08/2012  06/12/2012  02/05/2013 |
| **1819** | c.500A>G | c.364del | - | **-** | Asper LCA Array  AGRF RPE65 sequencing  AGRF AIPL1 sequencing  **CEI LCA Panel – 19 genes** | 24/06/2013  12/08/2013  12/08/2013  **13/02/2014** |
| **1965** | c.364del | c.769G>A | c.364del | WT | **CEI LCA Panel – 19 genes** | **30/09/2014** |
| **Protein chaperones and trafficking** | | | | | | |
| ***AIPL1* (NM_014336.3)** | | | | | | |
| 2030 | c.356_359del^BD^ | c.834G>A | c.356_359del | c.834G>A | **CEI RD Panel v8** | **03/08/2015** |
|  |  |  | c.356_359del | WT |  |  |
|  |  |  | WT | WT |  |  |
| 2272 | c.834G>A**^BD^** | c.277-2A>G | c.834G>A | c.277-2A>G | **CEI RD Panel v8** | **03/08/2015** |

Pedigree ID in Bold indicates simplex case. AGRF = Australian Genome Research Facility; ARRP = autosomal recessive retinitis pigmentosa; BD = by default (parental DNA not available but variant detected in related family member); CEI = Casey Eye Institute; DNWTP = did not wish to participate; LCA = Leber congenital amaurosis; NT = not tested; RD = retinal dystrophy; STGD/Mac Dys = Stargardt disease/macular dystrophy; WT = wildtype alelle. All heterozygous and homozygous WT individuals were unaffected. **The testing methodologies progressively employed for each pedigree are shown, with those denoted in bold resolving the primary cause of disease.**
