# Supplementary material for: The genetic profile of Leber congenital amaurosis in an Australian cohort
Source: Mol Genet Genomic Med. 2017 Aug 22;5(6):652–67. doi: 10.1002/mgg3.321 (PMC5702575; doi:10.1002/mgg3.321)
Supplement: Supplementary file 3 — Table S2. Pathogenicity data for genetic variants that account for or are likely to account for disease in this cohort, displayed by variant subtype. [file MGG3-5-652-s003.pdf]

Supplementary Table 2: Pathogenicity data for genetic variants that account for or are likely to account for disease in this cohort, displayed by variant subtype.

Insertion / Deletion Variants	Mutation Taster			SIFT INDEL				VEST INDEL		ExAC		Literature		
Nucleotide/ protein changes	Assignmentand Score	PhyloP/ PhastCons	Causes NMD	Effect	Confidence score	Causes NMD	(%) Indel Location	Score	p Value	MAF	Homozygotes / Allele number	Occurrence	Functional studies	Comments
AIPL1 **(NM_014336.3)**														
c.356_359del p. His119Argfs*31	P 1.00	4.006; 4.835/ 1.000	Y	Dam	0.858	Y	31	P 0.848	0.001	No Data		-	-	**Novel**
CEP290 **(NM_025114.3)**														
c.3175dup p. Ile1059Asnfs*11	P 1.00		Y	Dam	0.858	Y	43	P 0.745	0.001	0.0003	0 / 113,552	[1-3]	-	-
c.3181_3182del p. Met1061Alafs*8	P 1.00	3.314; 3.237/ 1.000	Y	Dam	0.858	Y	43	P 0.787	0.001	No Data		[4]	-	-
c.4625_4626insCATG p. Ala1543Metfs*37	P 1.00		Y	Dam	0.858	Y	62	P 0.841	0.001	No Data		-	-	**Novel**
CRB1 **(NM_201253.2)**														
c.613_619del p. Ile205Aspfs*13	P 1.00		Y	Dam	0.858	Y	14	P 0.756	0.000	No Data		[5-7]	-	-
c.1793del p. Pro598Hisfs*23	P 1.00	0.999/ 0.098	Y	Dam	0.858	Y	42	P 0.861	0.000	No Data		-	-	**Novel**
GUCY2D **(NM_000180.3)**														
c.91dup p. Arg31Profs*288	P 1.00		Y	Dam	0.579	Y	3	B 0.214	0.372	No Data		[8]	-	-
c.2516del p. Thr839Argfs*27	P 1.00	5.69/ 1.000	Y	Dam	0.858	Y	76	P 0.731	0.001	No Data		[9]	-	-
c.2595del p. Lys866Argfs*14	P 1.00	1.564/ 1.000	Y	Dam	0.858	Y	78	P 0.913	0.000	No Data		[4, 10]	-	-

LCA5 **(NM_001122769.2)**													
c.1144_1147dup<br>p. Asn383Thrfs*15	P<br>1.00		Pos	Dam	0.858	N	55	B<br>0.167	0.074	No Data	-	-	**Novel**
NMNAT1 **(NM_022787.3)**													
c.364del<br>p. Arg122Glyfs*20	P<br>1.00	2.822/<br>0.613	Pos	Dam	0.858	N	43	P<br>0.666	0.003	No Data	[11]	-	-
RPE65 **(NM_000329.2)**													
c.951_956del<br>p. Tyr318_Glu319del	pm<br>1.00	1.000	N	Dam	0.894	N	59	P<br>1.000	0.000	No Data	-	-	**Novel**
RPGRIP1 **(NM_020366.3)**													
**c.exon19del; chr14: g.(21798302_21798377)_(21798551_21799045) del (hg19,NC000014.8)**	P<br>1.00		Y	No Data				No Data		No Data	-	-	**Novel;**<br>Exon 17 deletion considered pathogenic for LCA [12]
TULP1 **(NM_003322.3)**													
c.524dup<br>p. Pro176Thrfs*7	P<br>1.00		Y	Dam	0.858	Y	32	B<br>0.418	0.052<br>borde<br>rline	No Data	-	-	**Novel**

Nonsense Variants	Mutation Taster					ExAC		Literature		Comments
Nucleotide/ protein changes	Assignment and Score	PhyloP/ phastCons	Causes NMD	Position (AA) of stop codon		MAF	Homozygotes / Alelle number	Occurrence	Functional studies	
				wt	mut					
AIPL1 **(NM_014336.3)**										
c.834G>A<br>p. Trp278*	P<br>1.00	4.931/<br>1.000	?	385	278	0.0004	0<br>119,090	[13-15]	[16]	-
CEP290 **(NM_025114.3)**										
c.1781T>A<br>p. Leu594*	P<br>1.00	0.447/<br>0.468	Y	2480	594	0.0001	0<br>27,218	[4]	-	-
c.2991+1655A>G#<br>p. Cys998*	pm<br>1.00	0.983/<br>0.109	Y	-	-	No Data		[17]	-	-
GUCY2D **(NM_000180.3)**										
c.2646C>G<br>p. Tyr882*	P<br>1.00	0.754/<br>1.000	Y	1104	882	No Data		-	-	**Novel**
NMNAT1 **(NM_022787.3)**										
c.507G>A<br>p. Trp169*	P<br>1.00	6.004/<br>1.000	?	280	169	0.0000	0<br>121,352	[11, 18, 19]	-	-

RDH12 **(NM_152443.2)**										
c.316C>T<br>p. Arg106*	P<br>1.00	2.018/<br>1.000	Y	317	106	0.0000	0<br>121,380	[20]	-	-
RPE65 **(NM_000329.2)**										
c.130C>T<br>p. Arg44*	P<br>1.00	2.687/<br>1.000	Y	534	44	0.0000	0<br>121,406	-	[21, 22]	-
RPGRIP1 **(NM_020366.3)**										
c.1219C>T<br>p. Gln407*	P<br>1.00	1.128/<br>0.598	Y	1287	407	0.0000	0<br>49,320	-	-	+1763-8C>G-NMD
c.1447C>T<br>p. Gln483*	P<br>1.00	0.451/<br>0.000	Y	1287	483	0.0000	0<br>119,622	[23]	-	(Referenced paper only found one heterozygous variant in RPGRIP1)
c.2935C>T<br>p. Gln979*	P<br>1.00	1.473/<br>0.016	Y	1287	979	No Data		-	-	**Novel**
SPATA7 **(NM_018418.4)**										
c.763C>T<br>p. Gln255*	P<br>1.000	2.614/<br>1.000	Y	600	255	No Data		[24]	[25]	-
TULP1 **(NM_003322.3)**										
c.1081C>T<br>p. Arg361*	P<br>1.000	1.101/<br>0.999	Y	543	361	No Data		[26, 27]	-	-

Missense Variants	Mutation Taster			Align GVGD		PolyPhen2		SIFT			ExAC		Literature		
**Nucleotide / protein changes**	**Assignment and Score**	**PhyloP/ phastCons**	**Grantham Score**	**Class**	**GV/GD**	**Hum Div**	**Hum Var**	**Pred**	**Score**	**Med Seq Cons**	**MAF**	**Homozygotes / Allele number**	**Occurrence**	**Functional studies**	**Comments**
CRB1 **(NM_201253.2)**															
c.2843G>A p. Cys948Tyr	P 1.00	5.31/ 1.00	194	C15	138.77 112.21	PD 1.00	PD 0.92	Del	0.04	2.69	0.0002	0 / 119,820	[4, 6, 28-36]	-	-
GUCY2D **(NM_000180.3)**															
c.307G>A p. Glu103Lys	pm 0.65	2.78/ 0.74	56	C0	240.48 0.00	PD 1.00	PD 0.99	Tol	0.29	3.22	No Data		[9, 37-39]		-
c.2302C>T p. Arg768Trp	P 1.00	3.75/ 1.00	101	C0	241.31 94.79	PD 1.00	PD 1.00	Del	0.00	3.22	0.0001	0 / 121,264	[8, 40-44]	[45, 46]	-
c.2345T>A p. Leu782His	pm 1.00	0.87/ 1.00	99	C0	258.55 0.00	B 0.00	B 0.00	Tol	0.29	3.22	0.1303	1537 / 121,268	-	-	**Benign**
c.2383C>T p. Arg795Trp	P 1.00	2.35/ 0.98	101	C0	241.31 94.79	PD 1.00	PD 1.00	Del	0.00	3.22	0.0001	0 / 121,100	[47, 48]	-	[43, 49, 50] indicate that other variants at this codon position are considered pathogenic for LCA

NMNAT1 **(NM_022787.3)**															
c.500A>G<br>p. Asn167Ser	P<br>1.00	3.45/<br>1.00	46	C0	217.79<br>0.00	PD<br>0.82	B<br>0.30	Tol	0.08/<br>9	3.12	No Data		-	-	**Novel**
c.769G>A<br>p. Glu257Lys	P<br>1.00	3.87/<br>1.00	56	C0	118.33<br>21.98	B<br>0.09	B<br>0.03	Tol	0.25/<br>7	3.11	0.0006	0<br>120,500	[11, 18, 19, 51, 52, 53]	[19, 54]	Incomplete penetrance/ Hypo-morphic allele
RDH12 **(NM_152443.2)**															
c.697G>C<br>p. Val233Leu	P<br>1.00	6.21/<br>1.00	32	C0	134.16<br>4.86	PD<br>0.96	PD<br>0.74	Tol	0.22	2.87	0.0000	0<br>120,420	[20, 55]	-	-
RPE65 **(NM_000329.2)**															
c.1040G>C<br>p. Arg347Pro	P<br>1.00	4.11/<br>1.00	103	C15	56.64<br>66.83	PD<br>1.00	PD<br>0.99	Del<br>(Low conf)	0.02	3.30	No Data<br>But Arg347His = 0.000008295		-	-	**Novel**
RPGRIP1 **(NM_020366.3)**															
c.1639G>T<br>p. Ala547Ser	pm<br>0.97	0.39/<br>0.95	99	C0	244.82<br>0.00	PD<br>1.00	PD<br>1.00	Tol	0.59	2.80	0.2105	2911<br>117,052	[56-61]	-	**Benign**

Splicing Variants	Mutation Taster			Spliceman		Other nomenclature	ExAC		Literature		Comments
**Nucleotide/ protein changes**	**Assignment and Score**	**PhyloP**	**PhastCons**	**L1 Distance**	**Ranking (%)**		**MAF**	**Homozygotes / Allele number**	**Occurrence**	**Functional Studies**	
***AIPL1* (NM_014336.3)**											
c.277-2A>G	Consensus Dinucleotides					IVS2-2A>G	0.00002	0 / 120,944	[25, 62]	[63]	-
***CEP290* (NM_025114.3)**											
c.297+3A>G	P 1.000	2.768	1.000	33753	61	-	No Data		-	-	**Novel**
c.5587-1G>C	Consensus Dinucleotides					IVS40 as G-C-1	No Data		[25, 35, 64]	-	-
***RPE65* (NM_000329.2)**											
c.726-1G>A	Consensus Dinucleotides					-	No Data		-	-	**Novel**
***TULP1* (NM_003322.3)**											
c.999+5G>C	P 0.999	1.463	0.782	37074	78	IVS10 ds G-C +5	No Data		[65, 66]	-	-

Abbreviations: **Mutation Taster**; P = pathogenic, pm = polymorphism, NMD = nonsense-mediated decay: **SIFT INDEL**; Dam = damaging: **VEST INDEL**; P = pathogenic, B = benign: **ExAC**; MAF = minor allele frequency; **PolyPhen2**; PD = probably damaging, B = benign: **SIFT**; Del = deleterious, Tol = tolerated, Med Seq Cons = median sequence conservation.

References:

1. Chaki M, Hoefele J, Allen SJ, Ramaswami G, Janssen S, Bergmann C, Heckenlively JR, Otto EA, and Hildebrandt F, 2011. Genotype-phenotype correlation in 440 patients with NPHP-related ciliopathies. Kidney Int. 80(11): p. 1239-45.
2. Sayer JA, Otto EA, O'Toole JF, Nurnberg G, Kennedy MA, Becker C, Hennies HC, Helou J, Attanasio M, Fausett BV, Utsch B, Khanna H, et al., 2006. The centrosomal protein nephrocystin-6 is mutated in Joubert syndrome and activates transcription factor ATF4. Nat Genet. 38(6): p. 674-681.
3. Yzer S, Hollander AI, Lopez I, Pott JW, de Faber JT, Cremers FP, Koenekoop RK, and van den Born LI, 2012. Ocular and extra-ocular features of patients with Leber congenital amaurosis and mutations in CEP290. Mol Vis. 18: p. 412-25.
4. Ellingford JM, Barton S, Bhaskar S, O'Sullivan J, Williams SG, Lamb JA, Panda B, Sergouniotis PI, Gillespie RL, Daiger SP, Hall G, Gale T, et al., 2016. Molecular findings from 537 individuals with inherited retinal disease. J Med Genet. 53(11): p. 761-767.
5. Bujakowska K, Audo I, Mohand-Saïd S, Lancelot M-E, Antonio A, Germain A, Léveillard T, Letexier M, Saraiva J-P, Lonjou C, Carpentier W, Sahel J-A, et al., 2012. CRB1 mutations in inherited retinal dystrophies. Human Mutation. 33(2): p. 306-315.
6. Corton M, Tatu SD, Avila-Fernandez A, Vallespin E, Tapias I, Cantalapiedra D, Blanco-Kelly F, Riveiro-Alvarez R, Bernal S, Garcia-Sandoval B, Baiget M, and Ayuso C, 2013. High frequency of CRB1 mutations as cause of Early-Onset Retinal Dystrophies in the Spanish population. Orphanet J Rare Dis. 8: p. 20.
7. van Huet RA, Pierrache LH, Meester-Smoor MA, Klaver CC, van den Born LI, Hoyng CB, de Wijs IJ, Collin RW, Hoefsloot LH, and Klevering BJ, 2015. The efficacy of microarray screening for autosomal recessive retinitis pigmentosa in routine clinical practice. Mol Vis. 21: p. 461-76.
8. Hanein S, Perrault I, Gerber S, Tanguy G, Barbet F, Ducroq D, Calvas P, Dollfus H, Hamel C, Lopponen T, Munier F, Santos L, et al., 2004. Leber congenital amaurosis: Comprehensive survey of the genetic heterogeneity, refinement of the clinical definition, and genotype-phenotype correlations as a strategy for molecular diagnosis. Human Mutation. 23(4): p. 306-317.
9. Stone EM, 2007. Leber congenital amaurosis - a model for efficient genetic testing of heterogeneous disorders: LXIV Edward Jackson Memorial Lecture. Am J Ophthalmol. 144(6): p. 791-811.
10. Jacobson SG, Cideciyan AV, Peshenko IV, Sumaroka A, Olshevskaya EV, Cao L, Schwartz SB, Roman AJ, Olivares MB, Sadigh S, Yau KW, Heon E, et al., 2013. Determining consequences of retinal membrane guanylyl cyclase (RetGC1) deficiency in human Leber congenital amaurosis en route to therapy: residual cone-photoreceptor vision correlates with biochemical properties of the mutants. Hum Mol Genet. 22(1): p. 168-83.
11. Perrault I, Hanein S, Zanlonghi X, Serre V, Nicouleau M, Defoort-Delhemmes S, Delphin N, Fares-Taie L, Gerber S, Xerri O, Edelson C, Goldenberg A, et al., 2012. Mutations in NMNAT1 cause Leber congenital amaurosis with early-onset severe macular and optic atrophy. Nat Genet. 44(9): p. 975-7.
12. Suzuki T, Fujimaki T, Yanagawa A, Arai E, Fujiki K, Wada Y, and Murakami A, 2014. A novel exon 17 deletion mutation of RPGRIP1 gene in two siblings with Leber congenital amaurosis. Jpn J Ophthalmol. 58(6): p. 528-35.

13. Aboshiha J, Dubis AM, van der Spuy J, Nishiguchi KM, Cheeseman EW, Ayuso C, Ehrenberg M, Simonelli F, Bainbridge JW, and Michaelides M, 2015. Preserved outer retina in AIPL1 Leber's congenital amaurosis: implications for gene therapy. Ophthalmology. 122(4): p. 862-4.
14. Sohocki MM, Bowne SJ, Sullivan LS, Blackshaw S, Cepko CL, Payne AM, Bhattacharya SS, Khaliq S, Qasim Mehdi S, Birch DG, Harrison WR, Elder FF, et al., 2000. Mutations in a new photoreceptor-pineal gene on 17p cause Leber congenital amaurosis. Nat Genet. 24(1): p. 79-83.
15. Sohocki MM, Daiger SP, Bowne SJ, Rodriguez JA, Northrup H, Heckenlively JR, Birch DG, Mintz-Hittner H, Ruiz RS, Lewis RA, Saperstein DA, and Sullivan LS, 2001. Prevalence of mutations causing retinitis pigmentosa and other inherited retinopathies. Human Mutation. 17(1): p. 42-51.
16. van der Spuy J, Chapple JP, Clark BJ, Luthert PJ, Sethi CS, and Cheetham ME, 2002. The Leber congenital amaurosis gene product AIPL1 is localized exclusively in rod photoreceptors of the adult human retina. Human Molecular Genetics. 11(7): p. 823-831.
17. den Hollander AI, Koenekoop RK, Yzer S, Lopez I, Arends ML, Voesenek KE, Zonneveld MN, Strom TM, Meitinger T, Brunner HG, Hoyng CB, van den Born LI, et al., 2006. Mutations in the CEP290 (NPHP6) gene are a frequent cause of Leber congenital amaurosis. Am J Hum Genet. 79(3): p. 556-61.
18. Chiang PW, Wang J, Chen Y, Fu Q, Zhong J, Chen Y, Yi X, Wu R, Gan H, Shi Y, Chen Y, Barnett C, et al., 2012. Exome sequencing identifies NMNAT1 mutations as a cause of Leber congenital amaurosis. Nat Genet. 44(9): p. 972-4.
19. Koenekoop RK, Wang H, Majewski J, Wang X, Lopez I, Ren H, Chen Y, Li Y, Fishman GA, Genead M, Schwartzentruber J, Solanki N, et al., 2012. Mutations in NMNAT1 cause Leber congenital amaurosis and identify a new disease pathway for retinal degeneration. Nat Genet. 44(9): p. 1035-9.
20. Mackay DS, Dev Borman A, Moradi P, Henderson RH, Li Z, Wright GA, Waseem N, Gandra M, Thompson DA, Bhattacharya SS, Holder GE, Webster AR, et al., 2011. RDH12 retinopathy: novel mutations and phenotypic description. Mol Vis. 17: p. 2706-16.
21. Pang JJ, Chang B, Hawes NL, Hurd RE, Davisson MT, Li J, Noorwez SM, Malhotra R, McDowell JH, Kaushal S, Hauswirth WW, Nusinowitz S, et al., 2005. Retinal degeneration 12 (rd12): a new, spontaneously arising mouse model for human Leber congenital amaurosis (LCA). Mol Vis. 11: p. 152-62.
22. Wright CB, Chrenek MA, Feng W, Getz SE, Duncan T, Pardue MT, Feng Y, Redmond TM, Boatright JH, and Nickerson JM, 2014. The Rpe65 rd12 allele exerts a semidominant negative effect on vision in mice. Invest Ophthalmol Vis Sci. 55(4): p. 2500-15.
23. Walia S, Fishman GA, Jacobson SG, Aleman TS, Koenekoop RK, Traboulsi EI, Weleber RG, Pennesi ME, Heon E, Drack A, Lam BL, Allikmets R, et al., 2010. Visual Acuity in Patients with Leber's Congenital Amaurosis and Early Childhood-Onset Retinitis Pigmentosa. Ophthalmology. 117(6): p. 1190-1198.
24. Perrault I, Hanein S, Gerard X, Delphin N, Fares-Taie L, Gerber S, Pelletier V, Merce E, Dollfus H, Puech B, Defoort-Dhellemmes S, Petersen MD, et al., 2010. Spectrum of SPATA7 mutations in Leber congenital amaurosis and delineation of the associated phenotype. Hum Mutat. 31(3): p. E1241-50.

25. Xiong HY, Alipanahi B, Lee LJ, Bretschneider H, Merico D, Yuen RK, Hua Y, Gueroussov S, Najafabadi HS, Hughes TR, Morris Q, Barash Y, et al., 2015. RNA splicing. The human splicing code reveals new insights into the genetic determinants of disease. Science. 347(6218): p. 1254806.
26. Glockle N, Kohl S, Mohr J, Scheurenbrand T, Sprecher A, Weisschuh N, Bernd A, Rudolph G, Schubach M, Poloschek C, Zrenner E, Biskup S, et al., 2014. Panel-based next generation sequencing as a reliable and efficient technique to detect mutations in unselected patients with retinal dystrophies. Eur J Hum Genet. 22(1): p. 99-104.
27. Guo Y, Prokudin I, Yu C, Liang J, Xie Y, Flaherty M, Tian L, Crofts S, Wang F, Snyder J, Donaldson C, Abdel-Magid N, et al., 2015. Advantage of Whole Exome Sequencing over Allele-Specific and Targeted Segment Sequencing in Detection of Novel TULP1 Mutation in Leber Congenital Amaurosis. Ophthalmic Genet. 36(4): p. 333-8.
28. Bernal S, Calaf M, Garcia-Hoyos M, Garcia-Sandoval B, Rosell J, Adan A, Ayuso C, and Baiget M, 2003. Study of the involvement of the RGR, CRPB1, and CRB1 genes in the pathogenesis of autosomal recessive retinitis pigmentosa. J Med Genet. 40(7): p. e89.
29. Cordovez JA, Traboulsi EI, Capasso JE, Sadagopan KA, Ganesh A, Rychwalski PJ, Neely KA, Brodie SE, and Levin AV, 2015. Retinal Dystrophy with Intraretinal Cystoid Spaces Associated with Mutations in the Crumbs Homologue (CRB1) Gene. Ophthalmic Genet. 36(3): p. 257-64.
30. de Castro-Miro M, Pomares E, Lores-Motta L, Tonda R, Dopazo J, Marfany G, and Gonzalez-Duarte R, 2014. Combined genetic and high-throughput strategies for molecular diagnosis of inherited retinal dystrophies. PLoS One. 9(2): p. e88410.
31. den Hollander AI, ten Brink JB, de Kok YJM, van Soest S, van den Born LI, van Driel MA, van de Pol DJR, Payne AM, Bhattacharya SS, Kellner U, Hoyng CB, Westerveld A, et al., 1999. Mutations in a human homologue of Drosophila crumbs cause retinitis pigmentosa (RP12). Nature Genetics. 23(2): p. 217-221.
32. Henderson RH, Mackay DS, Li Z, Moradi P, Sergouniotis P, Russell-Eggitt I, Thompson DA, Robson AG, Holder GE, Webster AR, and Moore AT, 2011. Phenotypic variability in patients with retinal dystrophies due to mutations in CRB1. Br J Ophthalmol. 95(6): p. 811-7.
33. Kousal B, Dudakova L, Gaillyova R, Hejtmankova M, Diblik P, Michaelides M, and Liskova P, 2016. Phenotypic features of CRB1-associated early-onset severe retinal dystrophy and the different molecular approaches to identifying the disease-causing variants. Graefes Arch Clin Exp Ophthalmol. 254(9): p. 1833-1839.
34. Riveiro-Alvarez R, Vallespin E, Wilke R, Garcia-Sandoval B, Cantalapiedra D, Aguirre-Lamban J, Avila-Fernandez A, Gimenez A, Trujillo-Tiebas MJ, and Ayuso C, 2008. Molecular analysis of ABCA4 and CRB1 genes in a Spanish family segregating both Stargardt disease and autosomal recessive retinitis pigmentosa. Mol Vis. 14: p. 262-7.
35. Yzer S, van den Born LI, Zonneveld MN, Lopez I, Ayyagari R, Teye-Botchway L, Mota-Vieira L, Cremers FP, and Koenekoop RK, 2007. Molecular and phenotypic analysis of a family with autosomal recessive cone-rod dystrophy and Stargardt disease. Mol Vis. 13: p. 1568-72.
36. Zaneveld J, Siddiqui S, Li H, Wang X, Wang H, Wang K, Li H, Ren H, Lopez I, Dorfman A, Khan A, Wang F, et al., 2015. Comprehensive analysis of patients with Stargardt macular dystrophy reveals new genotype-phenotype correlations and unexpected diagnostic revisions. Genet Med. 17(4): p. 262-70.

37. Borman AD, *A genotype-phenotype study of childhood onset retinal dystrophies*, in *Institute of Ophthalmology*. 2015, University College London: London.
38. Henderson RH, Waseem N, Searle R, van der Spuy J, Russell-Eggitt I, Bhattacharya SS, Thompson DA, Holder GE, Cheetham ME, Webster AR, and Moore AT, 2007. An assessment of the apex microarray technology in genotyping patients with Leber congenital amaurosis and early-onset severe retinal dystrophy. Invest Ophthalmol Vis Sci. 48(12): p. 5684-9.
39. Stone EM, 2003. Finding and interpreting genetic variations that are important to ophthalmologists. Trans Am Ophthalmol Soc. 101: p. 437-84.
40. Coppieters F, De Wilde B, Lefever S, De Meester E, De Rocker N, Van Cauwenbergh C, Pattyn F, Meire F, Leroy BP, Hellemans J, Vandesompele J, and De Baere E, 2012. Massively parallel sequencing for early molecular diagnosis in Leber congenital amaurosis. Genet Med. 14(6): p. 576-85.
41. Coppieters F, Lefever S, Leroy BP, and De Baere E, 2010. CEP290, a gene with many faces: mutation overview and presentation of CEP290base. Human Mutation. 31(10): p. 1097-1108.
42. Li L, Xiao X, Li S, Jia X, Wang P, Guo X, Jiao X, Zhang Q, and Hejtmancik JF, 2011. Detection of variants in 15 genes in 87 unrelated Chinese patients with Leber congenital amaurosis. PLoS One. 6(5): p. e19458.
43. Simonelli F, Ziviello C, Testa F, Rossi S, Fazzi E, Bianchi PE, Fossarello M, Signorini S, Bertone C, Galantuomo S, Brancati F, Valente EM, et al., 2007. Clinical and molecular genetics of Leber's congenital amaurosis: A multicenter study of Italian patients. Investigative Ophthalmology & Visual Science. 48(9): p. 4284-4290.
44. Yzer S, Leroy BP, De Baere E, de Ravel TJ, Zonneveld MN, Voesenek K, Kellner U, Ciriano JPM, de Faber J-THN, Rohrschneider K, Roepman R, den Hollander AI, et al., 2006. Microarray-Based Mutation Detection and Phenotypic Characterization of Patients with Leber Congenital Amaurosis. Investigative Ophthalmology & Visual Science. 47(3): p. 1167-1176.
45. Peshenko IV, Olshevskaya EV, Yao S, Ezzeldin HH, Pittler SJ, and Dizhoor AM, 2010. Activation of retinal guanylyl cyclase RetGC1 by GCAP1: stoichiometry of binding and effect of new LCA-related mutations. Biochemistry. 49(4): p. 709-17.
46. Zulliger R, Naash MI, Rajala RV, Molday RS, and Azadi S, 2015. Impaired association of retinal degeneration-3 with guanylate cyclase-1 and guanylate cyclase-activating protein-1 leads to leber congenital amaurosis-1. J Biol Chem. 290(6): p. 3488-99.
47. Pasadhika S, Fishman GA, Stone EM, Lindeman M, Zelkha R, Lopez I, Koenekoop RK, and Shahidi M, 2010. Differential macular morphology in patients with RPE65-, CEP290-, GUCY2D-, and AIPL1-related Leber congenital amaurosis. Invest Ophthalmol Vis Sci. 51(5): p. 2608-14.
48. Roman AJ, Cideciyan AV, Matsui R, Sheplock R, Schwartz SB, and Jacobson SG, 2015. Outcome measure for the treatment of cone photoreceptor diseases: orientation to a scene with cone-only contrast. BMC Ophthalmol. 15: p. 98.
49. Maranhao B, Biswas P, Gottsch AD, Navani M, Naeem MA, Suk J, Chu J, Khan SN, Poleman R, Akram J, Riazuddin S, Lee P, et al., 2015. Investigating the Molecular Basis of Retinal Degeneration in a Familial Cohort of Pakistani Decent by Exome Sequencing. PLoS One. 10(9): p. e0136561.

50. Zernant J, Kulm M, Dharmaraj S, den Hollander AI, Perrault I, Preising MN, Lorenz B, Kaplan J, Cremers FP, Maumenee I, Koenekoop RK, and Allikmets R, 2005. Genotyping microarray (disease chip) for Leber congenital amaurosis: detection of modifier alleles. Invest Ophthalmol Vis Sci. 46(9): p. 3052-9.
51. Bravo-Gil N, Mendez-Vidal C, Romero-Perez L, Gonzalez-Del Pozo M, Rodriguez-de la Rua E, Dopazo J, Borrego S, and Antinolo G, 2016. Improving the management of Inherited Retinal Dystrophies by targeted sequencing of a population-specific gene panel. Sci Rep. 6: p. 23910.
52. Falk MJ, Zhang Q, Nakamaru-Ogiso E, Kannabiran C, Fonseca-Kelly Z, Chakarova C, Audo I, Mackay DS, Zeitz C, Borman AD, Staniszewska M, Shukla R, et al., 2012. NMNAT1 mutations cause Leber congenital amaurosis. Nat Genet. 44(9): p. 1040-5.
53. Siemiatkowska AM, Schuurs-Hoeijmakers JH, Bosch DG, Boonstra FN, Riemslag FC, Ruiter M, de Vries BB, den Hollander AI, Collin RW, and Cremers FP, 2014. Nonpenetrance of the most frequent autosomal recessive leber congenital amaurosis mutation in NMNAT1. JAMA Ophthalmol. 132(8): p. 1002-4.
54. Sasaki Y, Margolin Z, Borgo B, Havranek JJ, and Milbrandt J, 2015. Characterization of Leber Congenital Amaurosis-associated NMNAT1 Mutants. J Biol Chem. 290(28): p. 17228-38.
55. Lee K, Berg JS, Milko L, Crooks K, Lu M, Bizon C, Owen P, Wilhelmsen KC, Weck KE, Evans JP, and Garg S, 2015. High Diagnostic Yield of Whole Exome Sequencing in Participants With Retinal Dystrophies in a Clinical Ophthalmology Setting. Am J Ophthalmol. 160(2): p. 354-363 e9.
56. Booij JC, Florijn RJ, ten Brink JB, Loves W, Meire F, van Schooneveld MJ, de Jong PT, and Bergen AA, 2005. Identification of mutations in the AIPL1, CRB1, GUCY2D, RPE65, and RPGRIP1 genes in patients with juvenile retinitis pigmentosa. J Med Genet. 42(11): p. e67.
57. Dryja TP, Adams SM, Grimsby JL, McGee TL, Hong DH, Li T, Andreasson S, and Berson EL, 2001. Null RPGRIP1 alleles in patients with Leber congenital amaurosis. Am J Hum Genet. 68(5): p. 1295-8.
58. Genomes Project C, Abecasis GR, Altshuler D, Auton A, Brooks LD, Durbin RM, Gibbs RA, Hurles ME, and McVean GA, 2010. A map of human genome variation from population-scale sequencing. Nature. 467(7319): p. 1061-73.
59. Hameed A, Abid A, Aziz A, Ismail M, Mehdi SQ, and Khaliq S, 2003. Evidence of RPGRIP1 gene mutations associated with recessive cone-rod dystrophy. J Med Genet. 40(8): p. 616-9.
60. Lupski JR, Reid JG, Gonzaga-Jauregui C, Rio Deiros D, Chen DC, Nazareth L, Bainbridge M, Dinh H, Jing C, Wheeler DA, McGuire AL, Zhang F, et al., 2010. Whole-genome sequencing in a patient with Charcot-Marie-Tooth neuropathy. N Engl J Med. 362(13): p. 1181-91.
61. Verma A, Perumalsamy V, Shetty S, Kulm M, and Sundaresan P, 2013. Mutational screening of LCA genes emphasizing RPE65 in South Indian cohort of patients. PLoS One. 8(9): p. e73172.
62. Sohocki MM, Perrault I, Leroy BP, Payne AM, Dharmaraj S, Bhattacharya SS, Kaplan J, Maumenee IH, Koenekoop R, Meire FM, Birch DG, Heckenlively JR, et al., 2000. Prevalence of AIPL1 mutations in inherited retinal degenerative disease. Mol Genet Metab. 70(2): p. 142-50.

63. Bellingham J, Davidson AE, Aboshiha J, Simonelli F, Bainbridge JW, Michaelides M, and van der Spuy J, 2015. Investigation of Aberrant Splicing Induced by AIPL1 Variations as a Cause of Leber Congenital Amaurosis. Invest Ophthalmol Vis Sci. 56(13): p. 7784-7793.
64. Perrault I, Delphin N, Hanein S, Gerber S, Dufier JL, Roche O, Defoort-Dhellemmes S, Dollfus H, Fazzi E, Munnich A, Kaplan J, and Rozet JM, 2007. Spectrum of NPHP6/CEP290 mutations in Leber congenital amaurosis and delineation of the associated phenotype. Hum Mutat. 28(4): p. 416.
65. Ajmal M, Khan MI, Micheal S, Ahmed W, Shah A, Venselaar H, Bokhari H, Azam A, Waheed NK, Collin RW, den Hollander AI, Qamar R, et al., 2012. Identification of recurrent and novel mutations in TULP1 in Pakistani families with early-onset retinitis pigmentosa. Mol Vis. 18: p. 1226-37.
66. den Hollander AI, Lopez I, Yzer S, Zonneveld MN, Janssen IM, Strom TM, Hehir-Kwa JY, Veltman JA, Arends ML, Meitinger T, Musarella MA, van den Born LI, et al., 2007. Identification of novel mutations in patients with Leber congenital amaurosis and juvenile RP by genome-wide homozygosity mapping with SNP microarrays. Invest Ophthalmol Vis Sci. 48(12): p. 5690-8.
