# Supplementary material for: The genetic profile of Leber congenital amaurosis in an Australian cohort
Source: Mol Genet Genomic Med. 2017 Aug 22;5(6):652–67. doi: 10.1002/mgg3.321 (PMC5702575; doi:10.1002/mgg3.321)
Supplement: Supplementary file 4 — Table S3. Variant information and interpretation. [file MGG3-5-652-s004.docx]

**Supplementary Table 3 – Variant Information and interpretation**

| mRNA | | Protein | | Coding Effect | Pathogenicity  Interpretation | Databases | | | Ped |
| --- | --- | --- | --- | --- | --- | --- | --- | --- | --- |
| Nucleotide change | **exon/**  **intron** | **Protein**  **change** | **Functional**  **Domain/region** |  |  | **LSDB/**  **LOVD/**  **ClinVar** | **dbSNP#** | **HGMD** |  |
| *AIPL1* (NM_014336.3) | | | | | | | | | |
| c.277-2A>G | i2 |  | | Splice site (exon 3 del) | Pathogenic | ✓ | rs140808549 | CS003285 | 1 |
| c.356_359del | 3 | **His119Argfs*31** | NMD | **Frameshift** | **Pathogenic** | **×** | **-** | **-** | 1 |
| c.834G>A | 6 | Trp278* | TPR3 repeat | Nonsense | Pathogenic | ✓ | rs62637014 | CM000003 | 2 |
| *CEP290* (NM_025114.3) | | | | | | | | | |
| c.297+3A>G | i5 |  | | **Splice site** | **VUS-SP** | **×** | **-** | **-** | 1 |
| c.1781T>A | 18 | Leu594* | NMD | Nonsense | Pathogenic | **×** | rs371496675 | - | 1 |
| c.2991+1655A>G | i26 | Cys998* | NMD | Nonsense^#^ | Pathogenic | ✓ | rs281865192 | CS064383 | 4 |
| c.3175dup | 28 | Ile1059Asnfs*11 | NMD | Frameshift | Pathogenic | ✓ | rs62640570 | CI062250 | 1 |
| c.3181_3182del | 28 | Met1061Alafs*8 | NMD | Frameshift | Pathogenic | **×** | **-** | **-** | 1 |
| c.4625_4626insCATG | 35 | **Ala1543Metfs*37** | NMD | **Frameshift** | **Pathogenic** | **×** | **-** | **-** | 1 |
| c.5587-1G>C | i40 |  | | Splice site | Pathogenic | ✓ | - | CS072147 | 1 |
| *CRB1* (NM_201253.2) | | | | | | | | | |
| c.613_619del | 2 | Ile205Aspfs*13 | NMD | Frameshift | Pathogenic | ✓ | rs62645752 | CD011132 | 1 |
| c.1793del | 6 | **Pro598Hisfs*23** | NMD | **Frameshift** | **Pathogenic** | **×** | **-** | **-** | 1 |
| c.2843G>A | 9 | Cys948Tyr | EGF-like 14 domain | Missense | Pathogenic | ✓ | rs62645748 (rs140074554) | CM992152 | 2 |
| *GUCY2D* (NM_000180.3) | | | | | | | | | |
| c.91dup | 2 | Arg31Profs*288 | NMD | Frameshift | Pathogenic | ✓ | rs61749663 | CI040950 | 1 |
| c.307G>A | 2 | Glu103Lys | Receptor, Ligand binding region | Missense | Pathogenic | ✓ | rs61749668 | CM077936 | 2 |
| c.2302C>T | 12 | Arg768Trp | Protein kinase domain | Missense | Pathogenic | ✓ | rs61750168 | CM004764 | 2 |
| c.2345T>A | 12 | Leu782His | Protein kinase domain | Missense | Benign | ✓ | rs8069344 (rs52837515/  rs61623443) | CM067679 | 1 |
| c.2383C>T | 12 | Arg795Trp | Protein kinase domain | Missense | Likely pathogenic | ✓ | rs765910207 | CM108136 | 1 |
| c.2516del | 13 | Thr839Argfs*27 | NMD | Frameshift | Pathogenic | **×** | rs756044745 | CD078241 | 1 |
| c.2595del | 14 | Lys866Argfs*14 | NMD | Frameshift | Pathogenic | **×** | - | CD130145 | 1 |
| c.2646C>G | 14 | **Tyr882*** | **NMD** | **Nonsense** | **Pathogenic** | **×** | **-** | **-** | 1 |
| *LCA5* (NM_001122769.2) | | | | | | | | | |
| c.1144_1147dup | 7 | **Asn383Thrfs*15** | - | **Frameshift** | **Pathogenic** | **×** | **-** | **-** | 1 |
| *NMNAT1* (NM_022787.3) | | | | | | | | | |
| c.364del | 4 | Arg122Glyfs*20 | Cytidyltransferase-like domain | Frameshift | Pathogenic | ✓ | - | CD127792 | 2 |
| c.500A>G | 5 | **Asn167Ser** | Cytidyltransferase-like domain | **Missense** | **VUS-SP** | **×** | **-** | **-** | 1 |
| c.507G>A | 5 | Trp169* | Substrate binding domain | Nonsense | Pathogenic | ✓ | rs371526758 | CM127758 | 1 |
| c.769G>A | 5 | Glu257Lys | C-terminal Helix | Missense | Pathogenic | ✓ | rs150726175 (rs386834258) | CM127755 | 2 |
| *RDH12* (NM_152443.2) | | | | | | | | | |
| c.316C>T | 5 | Arg106* | NMD | Nonsense | Pathogenic | **×** | rs752242512 | CM118724 | 1 |
| c.697G>C | 8 | Val233Leu | Glucose/ribitol dehydrogenase | Missense | Likely Pathogenic | **×** | rs140257538 | CM118723 | 1 |
| *RPE65* (NM_000329.2) | | | | | | | | | |
| c.130C>T | 3 | Arg44* | NMD | Nonsense | Pathogenic | **×** | rs368088025 | - | 1 |
| c.726-1G>A | i7 |  | | **Splice site** | **Pathogenic** | **×** | **-** | **-** | 1 |
| c.951_956del | 9 | **Tyr318_Glu319del** | **Carotenoid oxygenase** | **In-frame Deletion** | **VUS-SP** | **×** | - | - | 1 |
| c.1040G>C | 10 | **Arg347Pro** | **Carotenoid oxygenase** | **Missense** | **VUS-SP** | **×** | **-** | **-** | 1 |
| *RPGRIP1* (NM_020366.3) | | | | | | | | | |
| c.1219C>T | 10 | Gln407* | NMD | Nonsense | Pathogenic | **×** | rs775425686 | **-** | 1 |
| c.1447C>T | 11 | Gln483* | NMD | Nonsense | Pathogenic | **×** | rs368781265 | CM1010528 | 1 |
| c.1639G>T | 13 | Ala547Ser | Coiled coil | Missense | Benign | ✓ | rs10151259  (rs34263042/  rs52791984/  rs61722408/  rs386834254) | CM032029 | 1 |
| c.2935C>T | 18 | **Gln979*** | **NMD** | **Nonsense** | **Pathogenic** | **×** | **-** | **-** | 1 |
| c.exon19del; chr14:  g.(21798302_21798377)  _(21798551_21799045)  del (hg19,NC000014.8) | 19 | **Gln1034Argfs*6** | **NMD** | **Frameshift (exon 19 del)** | **Pathogenic** | **×** | **-** | **-** | 1 |
| *SPATA7* (NM_018418.4) | | | | | | | | | |
| c.763C>T | 6 | Gln255* | NMD | Nonsense | Pathogenic | **×** | - | CM101409 | 1 |
| *TULP1* (NM_003322.3) | | | | | | | | | |
| c.524dup | 6 | **Pro176Thrfs*7** | NMD | **Frameshift** | **Pathogenic** | **×** | **-** | **-** | 1 |
| c.999+5G>C | i10 |  | | Splice site | Likely Pathogenic | ✓ | - | CS076684 | 1 |
| c.1081C>T | 11 | Arg361* | NMD | Nonsense | Pathogenic | **×** | - | CM140477 | 1 |

Variants depicted in bold designate novel variants which, at the time of analysis, had not been reported in the literature or variant databases either as a cause of inherited retinal dystrophies or as a normal polymorphism; greyed cells denote where not applicable; ^#^ Nonsense variant – this variant represents an intronic change that results in the insertion of a cryptic exon in the mRNA and introduces a premature stop codon (den Hollander 2006); NMD = nonsense-mediated decay (functional domain/region therefore not applicable); Ped = number of pedigrees harbouring variant; VUS = variant of uncertain significance; SP = suspected pathogenic; TPR = tetratricopeptide repeat domain
