# Supplementary material for: The genetic profile of Leber congenital amaurosis in an Australian cohort
Source: Mol Genet Genomic Med. 2017 Aug 22;5(6):652–67. doi: 10.1002/mgg3.321 (PMC5702575; doi:10.1002/mgg3.321)
Supplement: Supplementary file 5 — Table S4. Comparison of available clinical or participant‐derived information displayed by functional category. [file MGG3-5-652-s005.pdf]

Supplementary Table 4 – Comparison of available clinical or participant-derived information displayed by functional category

ID	Genotype	Nystagmus	VA	ERG/VEP	Photophobia	Nyctalopia	Ref Err	Ant Seg Abn	Retinal fundus changes<br>Comments	Dx
**Ciliary transport and trafficking**										
CEP290 (NM_025114.3)										**OMIM #611755**
0623	c.4625_4626insCATG<br>c.2991+1655A>G	✓	NLP since birth	**0**^{10} Bilaterally flat ERG tracings					Roving nystagmus<br>Not drawn to bright lights<br>Eye poking<br>Attenuated retinal vessels	LCA
1015	c.2991+1655A>G<br>c.3181_3182delAT		NLP since birth					×	No fundi data	LCA
1212	c.2991+1655A>G<br>c.1781T>A	✓	LP since birth	**0**^{7} + **1**^{9} Bilaterally flat ERG tracings				×	Fundi normal at **0**^{7}	LCA
1576	c.5587-1G>C<br>c.3175dupA		LP since birth					K	Bilateral corneal grafts<br>Pigment spicules<br>Strong Bell's reflex precluded detailed retinal examination	LCA
1950<br>4434	c.2991+1655A>G<br>c.297+3A>G	✓	LP since birth						Eye poking<br>No fundi data	LCA
1950<br>5249	c.2991+1655A>G<br>c.297+3A>G	✓	LP now **17**		✓	×			Eye poking when younger<br>Coat's disease (**12-13**) exacerbated visual deficit<br>No fundi data	LCA
LCA5 (NM_001122769.2)										**OMIM #604537**
1894<br>4326	c.1144_1147dupCTAA<br>c.1144_1147dupCTAA	✓	LP since birth	**7**^{3}<br>Bilaterally flat ERG tracings	✓		×	K, C	Eye poking<br>Dense asteroid hyalosis^{OD}; Waxy looking optic disc^{OS}; attenuated retinal vessels; bone spicule pigment clumping (very marked parafoveally); generalised retinal thinning;<br>Congenital Descemet's membrane abnormalities	CRD
1894<br>4320	c.1144_1147dupCTAA<br>c.1144_1147dupCTAA		~2% at birth, now NLP	**16**^{7} bilaterally flat consistent with absent retinal function					Does not exhibit eye poking<br>Previously, was always drawn to light/experienced flashing lights/floating spots<br>No fundi data<br>Other condition: Underactive thyroid	FA

RPGRIP1 (NM_020366.3)										**OMIM #613826**
0479 0235	c.1219C>T;1763-8C>G c.exon19del; chr14: g.(21798302_21798377)_(21798551_21799045) del (hg19,NC000014.8)	✓	VA aided **3**[11] <6/60 **4**[1] ~6/60 **5**[5] <2/60	**3**[11] only VEP; Light Flash VEP Amp; no ERG **3**[11] reduced amp[BE] **4**[1] grossly abml[LE] **5**[5] flat ERG tracings bilaterally amblyopia?			H	×	Hypermetropia at 3 months **3**[11] reduced amplitudes-impaired conduction ONF-central vision **4**[1] no identifiable components recorded[OU]; abnormal small & large check VEP bilaterally; reduced conduction ONF-central, paracentral and peripheral vision[OS] **5**[5] flat rod, maximal rod-cone and flicker ERG tracings[OU] -severe retinal dysfunction; contrast sensitivity scores severely abnormal at all spatial frequencies in OS and most of OD - large check VEP impaired conduction ONF-paracentral vision[OD]; large check VEP showed better reproducibility than at **4**[1]; light flash VEP normal[OU]; Mother light sensitive No fundi data	LCA
0479 0233	c.1219C>T;1763-8C>G c.exon19del; chr14: g.(21798302_21798377)_(21798551_21799045) del (hg19,NC000014.8)	✓	**8**[5] <6/60	**8**[5] EOG grossly abnormal; Farnsworth-Munsell D15 colour test grossly abnormal; bilaterally flat ERG tracings				×	**8**[5] Unable to perform Arden Contrast sensitivity, Farnsworth 100 Hue and Pattern ERG tests; Mother light sensitive **8**[5] Pigmentary changes to fundi	LCA
1642 4913	c.1447C>T c.2935C>T	✓			✓	✓		×	No fundi data	LCA
1642 3675	c.1447C>T c.2935C>T	✓	**30** 6/60[LE] 6/48[RE]		✓	✓		×	Constricted fields of vision at **30** – 3°[OS] / 5°[OD] Can see sparks of gold/bright light continuously if concentrating No fundi data	LCA
SPATA7 (NM_018418.4)										**OMIM #604232**
1543 4058	c.763C>T c.763C>T	✓	LB since second decade		×	×		⟶	Corneal transplant at **27** improved vision No fundi data	LCA
1543 3424	c.763C>T c.763C>T	✓	LP since birth		×	×			More severe phenotype than sibling (above) No fundi data	LCA

TULP1 (NM_003322.3)										**OMIM #613843**
1620	c.524dupC<br>c.524dupC	✓	**17**[1] 6/60<br>**18**[1] 3/60		✓	✓	M		Big drop in vision at **12** and **15**[6]<br>**17**[1] consistent 10° vision all direction from central fixation; beyond that irregular/spotted areas<br>**18**[1] very little central vision left; blurred<br>No fundi data	LCA
2175<br>4820	c.999+5G>C<br>c.1081C>T	✓	**10**[3] 6/60		×	✓			Eye poking<br>Stares at bright light; central vision blurred; a little colour blind; no fundi data	LCA
2175<br>4821	c.999+5G>C<br>c.1081C>T	✓	**7** 6/120		×	✓			Eye poking<br>Stares at bright light; central vision blurred; vision worse than sibling (above); no fundi data	LCA
**Visual cycle**										
RDH12 (NM_152443.2)										**OMIM #612712**
1425	c.316C>T[NT]<br>c.697G>C[NT]	✓	**0** 5° PV<br>to<br>**38** LP		×	✓			Ophthalmic investigation at **0**[6] = rods and cones not developed properly, RPE pigment broken and scattered; strabismus and nyctalopia from birth; From **3** colour vision deteriorated; **8** could not see colour well; **13** no colour vision; vision stable until early **20**'s (became paler/washed out); lack of contrast; progression until **38** to LP in sunshine then blackness/like looking through fog	LCA
RPE65 (NM_000329.2)										**OMIM #204100**
1404	c.130C>T<br>c.951_956delCTATGA		LB since birth		×	✓			Has not seen ophthalmologist since late adolescence; could read large print at school; very light affected – completely night blind, prefers bright light; degree of sight depends on light level and task	LCA
1723	c.726-1G>A[NT]<br>c.1040G>C[NT]	✓	<6/120[BE]	**4**[3] ERG tracing bilaterally grossly abnormal	×	✓		×	**4**[3] ERG tracings show severely reduced response amplitudes<br>**4**[3] Pale optic nerve heads, upbeat nystagmus; MRI shows prominent optic nerve sheaths	LCA

**Phototransduction cascade**										
GUCY2D (NM_000180.3)										**OMIM #204000**
0535	c.307G>A<br>c.2595delG	✓	LP since birth	**0^{4}** Grossly abnormal ERG & VER tracings<br>**0^{11}** flat ERG tracings and abnormal VER				×	**0^{11}** no medial opacities<br>Faintly present direct pupillary responses<br>Fundi normal except for poor foveal reflex^{OU}	LCA
1836	c.2302C>T<br>c.2516delC	✓		**0^{7}** Flat ERG tracings (parent report)	×			×	Eye poking<br>Drawn to bright light<br>Fundi appear normal	LCA
2019	c.91_92insC<br>c.307G>A	✓	LP since birth	**0^{9}** Full field ERG – no reproducible responses (dim white flash, brighter white flash, 30Hz flicker)	×		HH	×	**0^{9}** Roving eye movements + intermittent nystagmus (horizontal or vertical); Drawn to bright light; sluggish pupillary responses<br>Eye poking<br>Fundi appear normal, with some abnormality in the reflection from the inner limiting membrane at high magnification	LCA
2123	c.2383C>T<br>c.2646C>G	✓	LP/ NLP since birth	**0^{6}** Cutaneous ERGS- severely depressed in amplitude bilaterally; VEP shows severely reduced visual function					No abnormality noted via retinal imaging	LCA
2599	c.2302C>T; 2345T>A<br>c.2302C>T; 2345T>A	✓	LP since birth				M		Eye poking<br>Drawn to light<br>Sluggish pupillary responses<br>No fundi data	LCA
**Cell – cell interaction**										
CRB1 (NM_201253.2)										**OMIM #613835**
0306<br>0459	c.1793delC<br>c.2843G>A	✓	**18^{4}**<br>HM/LP^{RE}<br>LP^{LE}	**18^{4}** bilaterally flat ERG tracings		✓		×	Disease progression gradual until **15-16**, then deterioration<br>**18^{4}** - bilateral pigmentary retinopathy; typical paramacular donut ring of vision; colour vision absent; normal but sluggish pupillary responses<br>**20** - depression	LCA
0306<br>0458	c.1793delC<br>c.2843G>A	✓	**5^{7}** 3/60<br>**8^{0}** 3/60 unaided,<br>5/60 aided	**5^{7}** + **8^{0}** bilaterally flat ERG tracings **5^{7}** Arden CST normal<br>**8^{0}** Arden CST total scores normal but individual abnormal		✓		×	**5^{7}** – small gliotic discs, pigmentary retinopathy, atrophic macula, arterioles attenuated, constricted fields, colour vision absent; pupillary responses sluggish; had bilateral surgical intervention for nystagmus;<br>Disease progression gradual till **15-17**, then deterioration; further stable until **23** then further	LCA

									deterioration; initially peripheral vision was worse than central vision but central vision has now deteriorated (**28**); uses what peripheral vision is left; reports blue-green colourblindness	
2274	c.613_619delATAGGAA<br>c.2843G>A	✓	**43**^{6} LP		✓	✓		C	Noticed deterioration by **30**. Has experienced peripheral and central vision loss, flashing lights and floating spots; has always worn glasses<br>No fundi data	LCA
**Neuroprotection**										
NMNAT1 (NM_022787.3)										**OMIM #608553**
1565	c.507G>A<br>c.769G>A	✓			✓				No fundi data	LCA
1819	c.364delA<br>c.500A>G	✓	LP since birth		✓				No fundi data	LCA
1965	c.364delA<br>c.769G>A	✓	NLP since birth		✓				No fundi data	LCA
**Protein chaperones and trafficking**										
AIPL1 (NM_014336.3)										**OMIM #604393**
2030<br>5551	c.355_358delCACA<br>c.834G>A		LP at birth; **~25** - NLP		×	×		K	Eye poking<br>Participant reports enophthalmia and corneal damage due to eye poking<br>Not drawn to light – used to see flashing lights in vision until deterioration to NLP in mid **20**'s<br>No fundi data – has not seen ophthalmologist since childhood (~**10**)	LCA
2030<br>5553	c.355_358delCACA<br>c.834G>A		LP since birth		×	×		×	Eye poking<br>No fundi data	LCA
2272<br>3117	c.277-2A>G<br>c.834G>A	✓	LP since birth					×	Eye poking<br>No fundi data	LCA
2272<br>3118	c.277-2A>G<br>c.834G>A	✓	LP since birth		×	×		×	Developmental delay<br>Symptoms worse than sibling (above)<br>Eye poking<br>Stared at bright lights when younger<br>No fundi data	LCA

Age reported = **years**^{months}**;** ✓ = present, × = not present, blank field = no data available;

Abbreviations: Ant Seg Abn, anterior segment abnormalities; C, cataract/s; CRD, cone-rod dystrophy; Dx, diagnosis; ERG, electroretinography; FA, fundus albipunctatus; H, hypermetropia; HH, high hypermetropia; HM, hand motion; K, keratoconus; LB, legally blind (VA≤6/60/visual field <10°); LCA, Leber congenital amaurosis; LP, light perception; M, myopia; NLP, no light perception; NT, not tested in parents (biallelism not determined); OD, oculus dexter (right eye); OMIM #, MIM Phenotype Number; OS, oculus sinister (left eye); OU, oculus uterque (both eyes); Ref Err, refractive errors; VA, visual acuity; VEP, vision evoked potentials.
